# The Hsp90 isoforms from *S. cerevisiae* differ in structure, function and client range

**DOI:** 10.1038/s41467-019-11518-w

**Published:** 2019-08-09

**Authors:** Hannah Girstmair, Franziska Tippel, Abraham Lopez, Katarzyna Tych, Frank Stein, Per Haberkant, Philipp Werner Norbert Schmid, Dominic Helm, Matthias Rief, Michael Sattler, Johannes Buchner

**Affiliations:** 1 Center for Integrated Protein Science at the Department of Chemistry, Technische Universität München, 85748 Garching, Germany; 2 Institute of Structural Biology, Helmholtz Zentrum München, 85764 Neuherberg, Germany; 3 Center for Integrated Protein Science at the Department of Physics, Technische Universität München, 85748 Garching, Germany; 4 Proteomics Core Facility, EMBL Heidelberg, 69117 Heidelberg, Germany

**Keywords:** Biochemistry, Biophysics, Cell biology, Structural biology, Chaperones

## Abstract

The molecular chaperone Hsp90 is an important regulator of proteostasis. It has remained unclear why *S. cerevisiae* possesses two Hsp90 isoforms, the constitutively expressed Hsc82 and the stress-inducible Hsp82. Here, we report distinct differences despite a sequence identity of 97%. Consistent with its function under stress conditions, Hsp82 is more stable and refolds more efficiently than Hsc82. The two isoforms also differ in their ATPases and conformational cycles. Hsc82 is more processive and populates closed states to a greater extent. Variations in the N-terminal ATP-binding domain modulate its dynamics and conformational cycle. Despite these differences, the client interactomes are largely identical, but isoform-specific interactors exist both under physiological and heat shock conditions. Taken together, changes mainly in the N-domain create a stress-specific, more resilient protein with a shifted activity profile. Thus, the precise tuning of the Hsp90 isoforms preserves the basic mechanism but adapts it to specific needs.

## Abstract (web summary)

*S. cerevisiae* encodes two Hsp90 isoforms, the constitutively expressed Hsc82 and stress-inducible Hsp82 that are 97% identical. Here, the authors combine a range of biophysical methods and show that they differ in their enzymatic properties, resilience to stress and client range, which suggests that they evolved to provide fine-tuned chaperone assistance under physiological and stress conditions.

## Introduction

Heat shock protein 90 (Hsp90) is an essential molecular chaperone in eukaryotes. Interference with its conformational cycle disrupts cellular function, as it is a regulator of proteins involved in cellular networks and signaling cascades^1^. Work over the last decades has revealed its structure and conformational transitions; however, we still lack a comprehensive picture of its biological roles^1,2^. Hsp90 consists of three domains: the ATP-binding N-terminal domain (NTD), the middle domain (MD), and the C-terminal domain (CTD). In addition, eukaryotic Hsp90 possesses a long charged linker between the NTD and the MD, which binds to the NTD, leading to a transient NTD-MD docked state^3^. The CTDs of two Hsp90 protomers associate and make Hsp90 a constitutive dimer, referred to as the “open state” of Hsp90. In addition to its C-terminal dimerization, the NTDs also undergo dimerization. This dimerization is a complex multistep process, in which the two NTDs dimerize and a β-strand is exchanged between them (closed 1 state) followed by the formation of the composite ATPase site by NTD/MD dimerization (closed 2 state)^2,4^. The transition to the closed 2 state and the relative dwell times in the open and closed conformations are important for client processing in vivo^2^. In line with this, the open and closed conformations are targeted by many of Hps90’s co-chaperones^5,6^.

Except for archaea and some bacteria, where Hsp90 is largely absent^7^, Hsp90 seems to be present in all organisms^7–9^. During evolution, gene duplications have led to variations in the Hsp90 isoform number among species^7,10,11^. Organelle-specific paralogues have evolved in protists, plants, and animals, which differ in mechanical properties and client specificity from the cytoplasmic isoform^12,13^. On an average, higher eukaryotes seem to contain more Hsp90 family members than lower ones^7^. *S. cerevisiae* possesses two cytoplasmic Hsp90 isoforms^8^, the cognate Hsc82 and the stress-inducible Hsp82. Under nonstress conditions, Hsc82 is expressed at tenfold higher levels than Hsp82. Heat shock only leads to a moderate induction of Hsc82 and a strong induction of Hsp82 such that the levels become equal^8^. Much of what we know about the Hsp90 machinery is based on work with yeast Hsp90. In most cases, the stress-induced isoform Hsp82 was used. Given that the isoforms share 97% sequence identity, it was assumed that they are identical.

Hsp90 works downstream of Hsp70 and has been suggested to interact with late folding intermediates^14^ or even fully folded proteins that have to be activated for association with different partners/ligands^15^. However, the structural determinants that underlie the interaction with Hsp90 have remained obscure. In mammals, clients typically belong to one of three protein families, E3 ligases, transcription factors, or kinases^16^. Different co-chaperones might contribute to this structural selection^17^. Moreover, metastability of the folds was suggested to be a central determinant for interaction with Hsp90 and would provide an explanation why some members of a certain protein family are clients while others are not^16^. For yeast, proteome-wide studies were mainly performed using chemical-genetic screens or synthetic genetic arrays. These screens found that Hsp90 is involved in a plethora of processes, but did not provide information about the structural basis of the interactions^18,19^.

Here, we systematically compared the yeast Hsp90 isoforms in terms of function and structural properties and determined their interactomes. Our analysis reveals surprising differences concerning stability, folding, enzymatic properties, and conformational regulation. We identify a large number of common client proteins, many of which were not identified so far, and a few isoform-specific clients. Together, our analysis suggests that the isoforms have evolved to provide fine-tuned chaperone assistance under physiological and stress conditions.

## Results

### Hsp82 and Hsc82 differ in their stabilities and folding

**Fig. 1:**
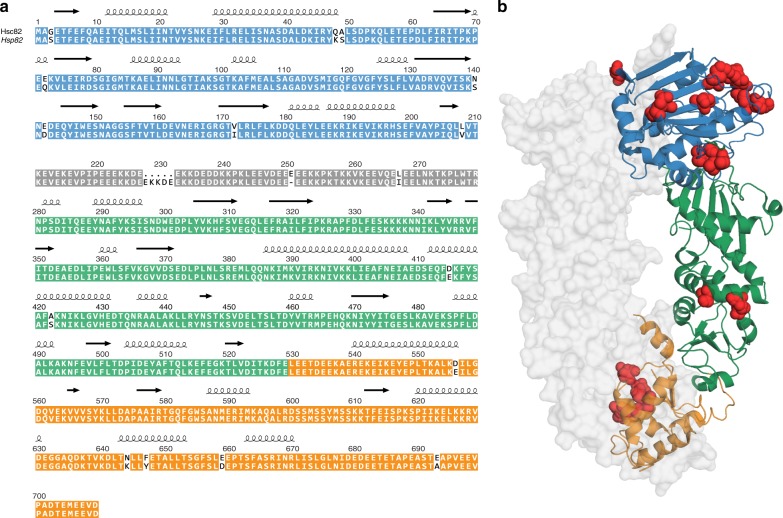
Comparison of amino acid sequence and structure of the yeast Hsp90 isoforms. **a** Sequence alignment of Hsc82 and Hsp82. The NTD of the isoforms is depicted in blue, the charged linker in gray, the MD in green, and the CTD in orange. The differences in amino acid sequence are highlighted in red. **b** Hsp82 structure in the closed state (PDB ID: 2CG9^20^). Sequence differences between Hsp82 and Hsc82 are highlighted in red

At the amino acid level, Hsp82 and Hsc82 of *S. cerevisiae* share 97% identity which corresponds to 16 differences in their amino acid sequences (Fig. 1a). In addition, the length of the charged linker between the NTD and the MD varies by four residues (Fig. 1a). The sequence differences cluster in the NTD (eight different residues) and CTD (five different residues), while only a single amino acid substitution is found in the linker and only two changes are present in the MD (Fig. 1a, b). The Hsp90 NTD, where most changes are located, consists of a twisted β-sheet covered on one face by α-helices^21^. Two helices (residues 28–50) and (residues 85–94) together with loop regions and residues that protrude from the β-sheet form a pocket for the nucleotide^21^ (Supplementary Fig. 1a). Upon nucleotide binding, a helical coil referred to as the “lid” closes over the nucleotide-binding pocket^20^. Neither the residues directly involved in binding to nucleotide nor water^21^ differ between Hsp82 and Hsc82. However, two amino acids that form part of the binding pocket (Q48K, A49S) and V172I, a residue next to the water-binding T171, differ (Supplementary Fig. 1a). In addition, S3 in Hsp82 is replaced by a glycine in Hsc82. S3 is part of the beta-strand that swaps over and forms hydrogen bonds with the other monomer in the N-terminally closed state (Fig. 1a, b)^20,22^.

To determine differences in structure and stability, Hsp82 and Hsc82 were expressed in *E. coli* and purified to homogeneity. CD spectroscopy indicated that no large structural alterations in the secondary structure exist (Supplementary Fig. 1b). The isoforms displayed differences in their thermal stabilities with melting temperatures of 60.4 ± 0.5 °C for Hsp82 and of 57.1 ± 0.2 °C for Hsc82 in the absence of a nucleotide (Table 1 and Supplementary Fig. 1c). In the presence of the slowly hydrolyzed ATP-analog ATPγS, which promotes the formation of the N-terminally closed conformation of Hsp90^4^, the melting temperature is increased by about 3 °C for both isoforms (Table 1 and Supplementary Fig. 1c). Thus, the stress-induced isoform is more stable than the constitutive one.

**Table 1:** Thermal stability of the Hsp90 isoforms and mechanical stability of their individual domains

Isoform	T_m_ (°C)	Domain	Expected length (nm)	Measured length (nm)	Unfolding force 1 (pN)	Unfolding force 2 (pN)	Unfolding force 3 (pN)
Hsp82	60.4 ± 0.5	Hsp82 NTD	71.8	69.5 ± 2.5	15.5 ± 1.6	12.1 ± 1.3	16.3 ± 2.2
Hsp82 + ATPγS	63.3 ± 0.4	Hsp82 MD	84.5	85.0 ± 1.5	19.2 ± 1.6		
Hsc82	57.1 ± 0.2	Hsp82 CTD	40.5	40.2 ± 1.5	11.1 ± 2.0		
Hsc82 + ATPγS	60.8 ± 0.4	Hsc82 NTD	71.8	66.8 ± 1.7	15.0 ± 1.9	10.2 ± 0.88	15.1 ± 2.6
		Hsc82 MD	84.5	83.9 ± 1.5	20.1 ± 1.8		
		Hsc82 CTD	40.5	42.3 ± 2.0	10.8 ± 1.9		

Melting temperatures (*T*_*m*_) of the Hsp90 isoforms were determined by tracking SYPRO orange binding upon thermal unfolding in the absence or presence of ATPγS. Means of three technical replicates and standard derivation are shown. To determine the mechanical stability of the Hsp90 domains of the isoforms optical trapping experiments were performed at 30 °C and the expected gains in contour length were compared with the measured gains in contour length. The indicated domains were pulled at different speeds and the average unfolding forces are indicated: Unfolding force 1: average unfolding force at 500 nm/s, unfolding force 2: average unfolding force at 20 nm/s, unfolding force 3: average unfolding force at 20 nm/s in the presence of 10 µM RD. Average forces and SD were calculated from sample sizes of: 426 events and 132 events for unfolding force 1 for Hsp82 and Hsc82, respectively; 82 events and 38 events for unfolding force 2 for Hsp82 and Hsc82, respectively; and 16 events and 34 unfolding events for unfolding force 3 for Hsp82 and Hsc82, respectively.

**Fig. 2:**
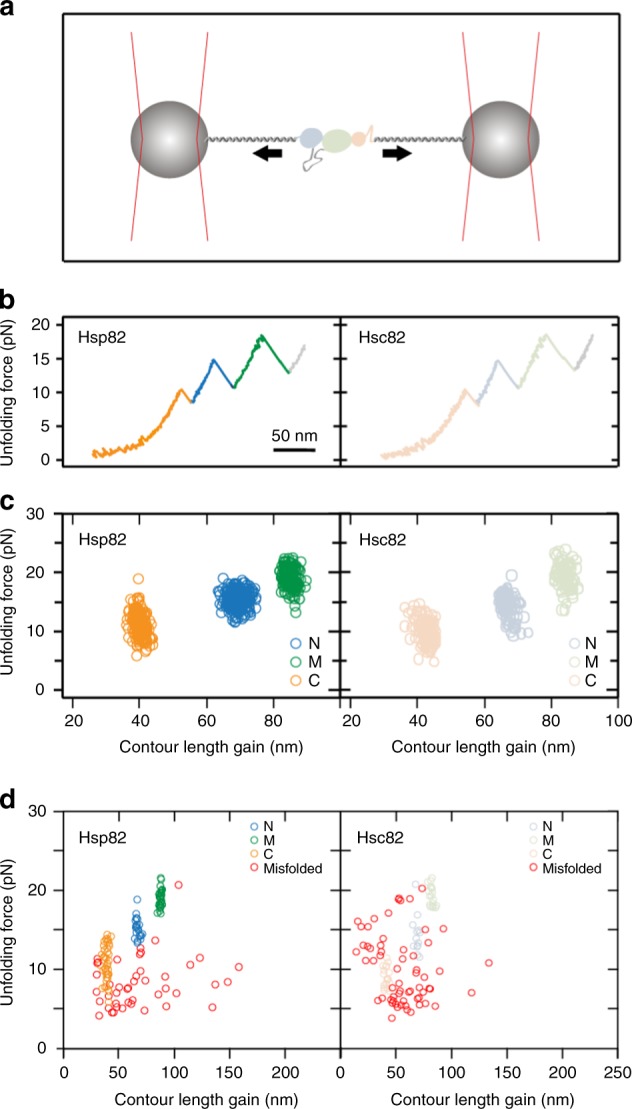
Unfolding of Hsp82 and Hsc82. **a** Schematic depicting how force is applied across the monomer of Hsc82 or Hsp82 using optical trapping (see Methods for details). **b** Example unfolding traces of Hsp82 (left) and Hsc82 (right) pulled at a constant velocity of 500 nm/s. The traces are colored according to domain. In both these example traces, the CTD is seen to unfold first (shown in orange), followed by the NTD (blue) and finally, the middle domain (green). **c** Performing repeated force-extension cycles and recording the unfolding forces and contour length gains for each domain results in the scatter plots shown here. The average unfolding forces and contour length gains for Hsp82 and Hsc82 are the same within error (see Table 1). **d** Repeated force-extension cycles at 500 nm/s with no waiting time at zero force result in large numbers of force-extension traces that do not show the native unfolding pattern. This occurs as a result of inter- and intra-domain misfolds in the monomers of Hsc82 and Hsp82, which is why misfolds with contour length gains longer than those of natively folded domains are common. Here, native mechanical signatures of individual domains are colored according to the domain (blue for the NTD, green for the middle domain, orange for the CTD), and events which did not match the native unfolding signatures for any domains are shown in red. Hsp82 data (left-hand side) is from 38 force-extension cycles for a single molecule, and Hsp82 data (right-hand side) is from 55 force-extension cycles for a single molecule

To explore the stabilities of Hsp82 and Hsc82 further, we performed single molecule optical trapping experiments (Fig. 2a). Typical force-extension unfolding traces for Hsp82 and Hsc82 monomers are shown in Fig. 2b. Three main unfolding events can be seen, each corresponding to one of the three domains. Recording the unfolding force and gain in contour length for each unfolding event in repeated force-extension cycles results in scatter plots such as those shown in Fig. 2c. For both isoforms, three separate clusters of unfolding events can be seen each corresponding to one domain. A detailed comparison of the resulting average gains in contour length and average unfolding forces for each domain of Hsp82 and Hsc82 is given in Table 1. In summary, the unfolding forces and contour length gains for each domain are the same for the two isoforms within error. Addition of ATP did not change the unfolding pattern of Hsc82 (unfolding forces with ATP for comparison with the data without ATP given in Table 1 are: *N*_hsc82_ = 15.8 ± 2.2, *M*_hsc82_ = 19.9 ± 1.4, *C*_hsc82_ = 10.3 ± 1.9). This was expected, as monomers not dimers are used in the optical trapping measurements. We next compared the refolding capabilities of the two isoforms by performing repeated force-extension stretch and relax cycles. Both successful refolding of individual domains (blue, green, and orange circles) as well as misfolding (red circles) was observed (Fig. 2d). Hsp82 refolded fully ~29% of the time (58 traces out of 200 cycles, four molecules), whereas Hsc82 was found to refold fully in ~14% of the events (42 traces out of 298 cycles, three molecules). Thus, the stress-induced isoform shows improved refolding compared with the constitutive isoform. We next used equilibrium measurements to characterize both the energetics and dynamics of the charged linker of Hsc82 and compared them with those previously measured for Hsp82^3^ (Supplementary Fig. 1d). The dynamics are described as the rate of transition between the docked state of the charged linker with a stable secondary structure and the undocked state^3^. We found that the charged linker of Hsc82 has a slightly greater free energy of stabilization than that of Hsp82 (1.43 kBT ± 0.3 for Hsc82 compared with that of 1.1 kBT ± 0.4 for Hsp82); however, this effect is small and within the experimental uncertainty of the measurement (Supplementary Fig. 1d).

### Hsp82 and Hsc82 differ in their ATPase activities

**Fig. 3:**
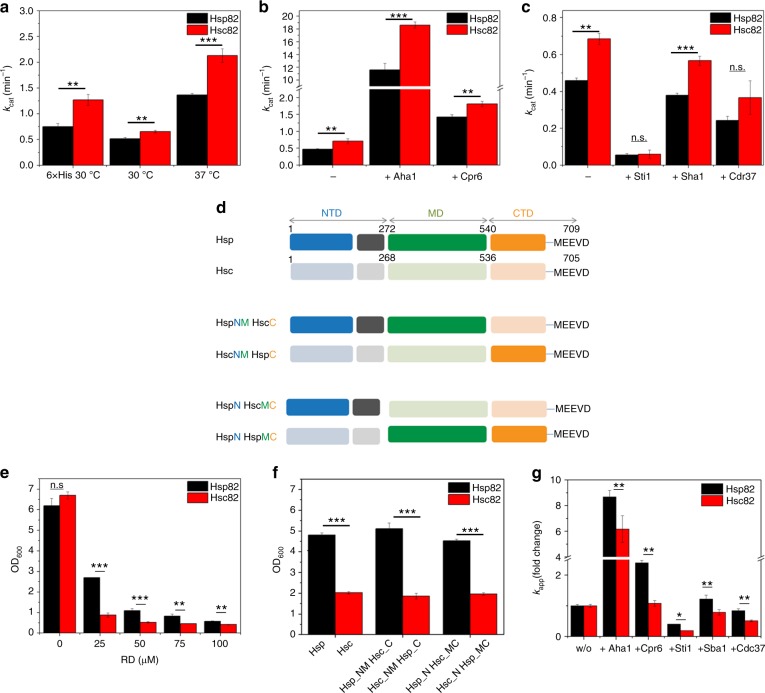
ATPases, RD sensitivity, and closing kinetics of yeast Hsp90 isoforms. For all measurements three technical replicates were used to determine standard deviations. Statistical significance was assessed using a two-sample *t*-test. The level of significance is indicated (ns: *p* > 0.05, *p < 0.05, **p < 0.01, ***p < 0.001. **a** Comparison of the ATPase activity of tagged (6×His) and untagged Hsp82 and Hsc82 at indicated temperatures. ATPase assays were performed in a standard buffer containing 2 mM ATP and a final concentration of 3 µM Hsp90. **b** The isoforms’ ATPase stimulation by Aha1 or Cpr6 was measured in a low salt buffer containing 40 mM HEPES, pH 7.5, 50 mM KCl, 5 mM MgCl_2_, 2 mM ATP and a final concentration of 1 µM Hsp82/Hsc82 or 3 µM Hsp82/Hsc82 in the presence of 30 µM Aha1 or 15 µM Cpr6, respectively. **c** The isoforms’ ATPase inhibition by Sti1, Sba1, and Cdc37 was measured in the low salt buffer containing a final concentration of 3 µM Hsc82/Hsp82 in absence of co-chaperone and in presence of 7.5 µM Sti1, 10 µM Sba1, and 20 µM Cdc37. **d** Scheme depicting Hsp90 chimeras used for RD assay shown in **e**. **e** Yeast expressing either Hsc82 or Hsp82 as the sole Hsp90 source was grown in the absence or presence of indicated concentration of the Hsp90 inhibitor RD. Yeast cell growth was measured after 20 h at OD_600_. **f** RD sensitivity of chimera of Hsp82 and Hsc82. The assay was performed as described in **e**. **g** Nucleotide-induced closing kinetics of Hsp82 and Hsc82 were recorded by FRET in the presence 2 mM ATPγS, in the absence of co-chaperone, or in the presence of the co-chaperone Aha1, Cpr6, Sti1, Sba1, Cdc37. The fold change (fc) in the closing kinetics constant (*k*_app_) in the presence of co-chaperones compared *k*_app_ in the absence of co-chaperones is shown for both isoforms

Since the presence of an N-terminal His-tag stimulates the ATPase activity of both isoforms (Fig. 3a and Table 2), we used proteins with native N-termini in the following. A comparison of the ATPase activities of Hsp82 and Hsc82 shows that the enzymatic activity of Hsc82 is ~1.3-fold higher than that of Hsp82 at 30 °C and ca. 1.6-fold higher at 37 °C (Fig. 3a and Table 2). Only a slight difference in the affinity for ATP binding was detected between the isoforms (Table 2). Thus, the differences in ATPase do not seem to result from differences in ATP binding but may be due to changes in conformational cycling.

**Table 2:** ATP hydrolysis rates of the isoforms

Isoform	Hsp82	Hsc82
ATPase (k_cat_ (min^−1^)) at 30 °C	0.52 ± 0.02	0.65 ± 0.02
with Aha1	11.59 ± 1.05 (≙2229%)	18.63 ± 0.48 (≙2866%)
with Cpr6	1.42 ± 0.07 (≙273%)	1.81 ± 0.08 (≙278%)
with Cdc37	0.24 ± 0.02 (≙46%)	0.37 ± 0.09 (≙57%)
with Sba1	0.38 ± 0.01 (≙73%)	0.57 ± 0.03 (≙88%)
with Sti1	0.06 ± 0.01 (≙12%)	0.06 ± 0.02 (≙9%)
ATPase (k_cat_ (min^−1^) at 37 °C	1.37 ± 0.02	2.13 ± 0.13
Isoform	6His-Hsp82	6His-Hsc82
ATPase (k_cat_ (min^−1^)) at 30 °C	0.75 ± 0.06	1.23 ± 0.10
Isoform domain	Hsp82NTD	Hsc82NTD
Affinity for ATP (K_d_ (µM))	88 ± 14	151 ± 33
Affinity for RD (K_d_ (nM))	2.7 ± 1.6	2.0 ± 0.8

The ATPase activities of Hsp82 and Hsc82 were measured with an ATP-regenerating system^23^. The influence of temperature (30 °C versus 37 °C), of 6His-tagging, and of co-chaperones on the ATPases are shown. In addition, affinities for ATP and RD were determined by ITC and *K*_d_ values are indicated.

We next explored how co-chaperones that modulate the ATPase of Hsp90^24^ affect the two isoforms. For the two co-chaperones that accelerate the ATPase activity of Hsp90 (Aha1 and Cpr6), we found that they stimulated the activity of both isoforms almost equally (Fig. 3b and Table 2). When we tested the three co-chaperones that inhibit Hsp90’s ATPase (Cdc37, p23/Sba1, Sti1), here too, only minor differences in the inhibitory effect were detected (Fig. 3c and Table 2). We next explored the binding affinities of the co-chaperones to the Hsp90 isoforms by analytical ultracentrifugation (AUC). The isoforms were labeled at C61 with ATTO 488. All co-chaperones bound to labeled Hsp90 except for Cdc37. Binding of Cdc37 to Hsp90 was thus explored with ATTO 488 labeled Cdc37 (Supplementary Fig. 2a). All co-chaperones displayed similar affinities for the two Hsp90 isoforms. However, for complexes with Cpr6 and p23/Sba1 differences in the *s*-values and fluorescence intensities were detected, suggesting that these two co-chaperones display differences in their binding mode (Supplementary Fig. 2a).

### Differences in growth and inhibitor sensitivity

**Fig. 4:**
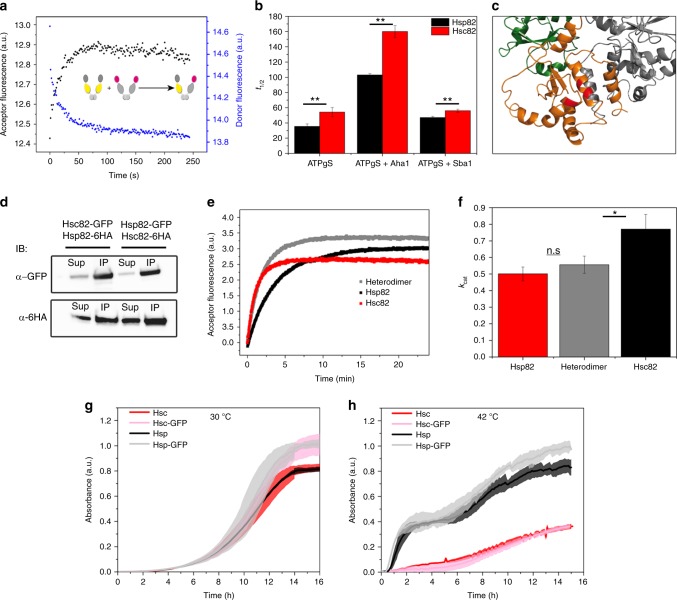
Subunit exchange, closed state stability, and heterodimerization. **a** Scheme depicting FRET experiment in the absence of a nucleotide used to determine the subunit exchange rate *k*_se_. **b** NTD stability of Hsp82 and Hsc82 was investigated by FRET chase experiments. The chase was induced by adding a tenfold excess of unlabeled Hsp90 to closed Hsp90 FRET complexes that were performed in the presence of 2 mM ATPγS. The apparent half-lives of the complexes were determined in the absence of co-chaperones or in the presence of Aha1 or Sba1. Three technical replicates were used to determine standard deviations. Statistical significance was assessed using a two-sample *t*-test. The level of significance is indicated (ns: *p* > 0.05, *p < 0.05, **p < 0.01, ***p < 0.001. **c** Cartoon representation of the dimerized CTD of Hsp82. Differences in amino acid sequence between the yeast Hsp90 isoforms are highlighted in red. **d** Yeast strains in which one Hsp90 isoform was GFP-tagged and the other isoform was HA-tagged were used to investigate heterodimerization. Co-immunoprecipitations were performed with an anti-GFP antibody. Western blots were developed with anti-GFP and anti-HA antibodies to determine the fraction of the HA-tagged isoform that co-immunoprecipitates with the GFP-tagged isoform. The supernatant fraction and the co-immunoprecipitated fraction are indicated. **e** Nucleotide-induced kinetics of Hsp82, Hsc82, and a heterodimer between Hsp82 and Hsc82 followed by FRET in the presence of ATPγS. The increase in acceptor fluorescence signal was followed and fitted to a mono-exponential function to obtain the apparent rate constants *k*_app_. **f** Comparison of the ATPase activities of Hsp90 isoforms and the heterodimer. Experiments were performed as described in Fig. 3a. Three technical replicates were used to determine standard deviations. The *k*_cat_ of the heterodimer was compared with the *k*_cat_ of Hsc82 and Hsp82 using a two-sample *t*-test. The level of significance is indicated (ns: *p* > 0.05, *p < 0.05, **p < 0.01, ***p < 0.001). **g**, **h** Yeast expressing plasmid-encoded Hsp90 isoforms (from p423GFP plasmids) or their GFP-tagged counterparts (from p425GPD plasmids) were compared in their growth at 30 or 42 °C in rich medium. Standard deviations are derived from three biological replicates

To examine whether the two isoforms differently impact growth when expressed as the sole source of Hsp90, we introduced plasmids expressing either Hsp82 or Hsc82 via a shuffling approach into the hsp82/hsc82 deletion strain. The isoforms were expressed at similar levels (Supplementary Fig. 3a). We recorded growth curves for the strains at different temperatures. At 30 °C no difference in growth rates was observed between the isoforms. However, under heat shock conditions (42 °C), yeast expressing Hsp82 grew better than those expressing Hsc82 (Fig. 4g, h).

We then tested if the isoforms differ in their sensitivity towards the inhibitor radicicol (RD). RD is a macrocyclic compound, which binds to the nucleotide binding pocket of Hsp90 and interferes with p23/Sba1 binding and client maturation^25^. In vitro, the affinities of the isolated NTDs of Hsp82 and of Hsc82 for RD turned out to be similar (Table 2) as determined by isothermal titration calorimetry (ITC) measurements. Also, the addition of RD had a comparably strong effect on the mechanical stability of the NTDs of both Hsp82 and Hsc82, as measured by the unfolding force (Table 1 and Supplementary Fig. 1e), but no effect on in the MD and CTD was observed (see for example Supplementary Fig. 1e, left panel). To test the effect of RD in vivo we used the above described shuffling strains. Yeast expressing Hsc82 were more strongly affected by RD than Hsp82, in particular in the lower concentration range (Fig. 3e): at 25 µM RD, yeast expressing Hsc82 showed a more than 2.5 times stronger inhibition of their growth than cells expressing Hsp82, in line with what has been previously described^26^. To obtain domain-specific information, we constructed chimera of Hsp82 and Hsc82 (Fig. 3d) and shuffled them into yeast as the sole Hsp90 source. All these Hsp90 variants supported viability and were expressed at equal levels (Supplementary Fig. 3a). The chimera revealed that the higher susceptibility of Hsc82 to RD exclusively relies on its NTD as the effect could be reconstituted when the Hsc82 NTD was transplanted onto Hsp82 (Fig. 3f).

### Conformational differences and binding of ligands

**Fig. 5:**
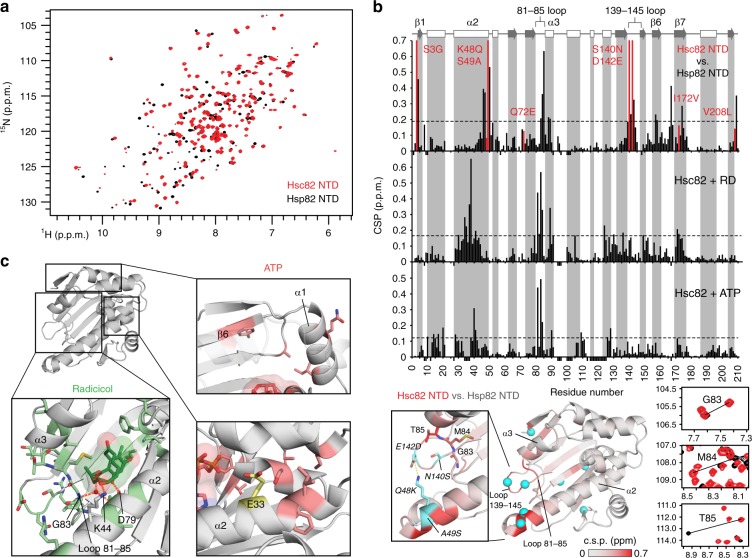
Comparison of the NTDs of yeast Hsp90 isoforms by NMR. **a** Overlay of ^1^H-^15^N HSQC spectra of Hsc82 (red) and Hsp82 (black). **b** Chemical shift perturbation (CSP) of apo-Hsc82 vs. apo-Hsp82 (top), Hsc82+RD (middle), and Hsc82+ATP (bottom). Red bars indicate residues that differ between the two isoforms, negative bars represent residues that are missing. Secondary structure elements derived from NMR secondary chemical shifts using TALOS+ are shown on top (arrow: β-strand: rectangles: α-helix). The CSP comparing isoforms are mapped onto the crystal structure of Hsp82 NTD (PDB id 1AH6^27^, bottom), with isoform-specific residues shown as cyan spheres. Inset shows a close-up view of the C-end of helix α2 and surrounding loops (bold), together with the salt bridge between Lys48 and Asp142 (italic). Panels at the right are zoomed views of the spectra in **a** showing peak shifts from residues of the 81–85 loop. **c** Mapping of differential CSPs of the two isoforms upon binding of RD (green sticks) and ATP (red sticks). For RD, regions with higher deviations correspond to loop 81–85, helixes α2, and α3 (bold), while for ATP to strand β6 and helix α1, together with residues surrounding the catalytic residue Glu33

Given the isoform-specific differences in the ATPase and sensitivity towards RD, we compared the structural properties of the NTD using NMR spectroscopy. As seen in Fig. 5a, the ^1^H,^15^N heteronuclear single quantum coherence (HSQC) NMR spectra of the two isoforms are highly similar, indicating that the overall structures of the NTDs are conserved. This notion is supported by the analysis of ^13^C secondary chemical shifts, which demonstrate that the NTDs share the same secondary structure (Supplementary Fig. 5a). However, several NMR signals show significant chemical shift differences. To analyze these alterations in more detail, we calculated the chemical shift perturbation (CSP), i.e., chemical shift differences of the backbone amide NMR signals of the two isoforms. Interestingly, apart from positions adjacent to residues that are different in the two isoforms, significant CSPs appear in additional allosteric regions (Fig. 5b, top). When plotted on the structure, most of these regions cluster at the C-terminal end of helix α2 and loop 139–145, extending to the neighboring 81–85 loop and helix α3 near the binding pocket (Fig. 5b, bottom). Interestingly, the C-terminal end of α2 and loop 139–145 contain four of the amino acid changes. One of them leads to a disruption of the salt bridge between Lys48 and Asp142 that is seen in the crystal structure of the Hsp82 NTD^27^.

To probe if the structural changes of Hsc82 NTD affect the binding of ATP and RD, we analyzed the CSPs caused by the addition of these compounds. CSPs with respect to Hsp82 binding were analyzed using the individual ^1^H and ^15^N chemical shift differences (Δδ). The respective CSP plots show differences in the binding pocket and the lid similar to those described previously for Hsp82^28^ (Fig. 5b, middle panels and Supplementary Fig. 4b). Analysis of ^1^H and ^15^N Δδs revealed several residues that deviate significantly between the isoforms (Supplementary Fig. 5). In general, more variations are seen for the RD-bound NTD, especially in the 81–85 loop, helix α3, and Phe124 and Lys173 from the binding pocket (Fig. 5c, lower left panel). Further differences appear in the middle of helix α2, around Lys44 and Asp79, two residues involved in hydrogen bonding with the inhibitor^29^. Regarding the ATP-bound isoforms, differences are also seen in Gly83 from the 81–85 loop, a residue that forms a hydrogen bond with nucleotides^29^. Nevertheless, in contrast to RD, most alterations appear far from the binding site, in particular affecting residues adjacent to the N-terminal strand β1 and helix α1, and Ile29, Phe30, Ser126 in the proximity of the catalytic residue Glu33^30^ (Fig. 5c, right panels). Our data thus suggest that RD binding slightly differs between the two isoforms. ATP seems to affect certain parts of the NTD that are far from the binding site. This indicates that changes in these regions could interfere with inter-domain contacts in the full-length protein and allosterically contribute to catalysis.

### Conformational changes and co-chaperone regulation differ

Hsp90 changes from an open, CTD-dimerized conformation to an additionally NTD-dimerized, closed conformation during its ATPase cycle. We wondered whether the differences in the NTDs affect the closing kinetics. Hsp90’s transition to the closed conformation can be tracked by a Förster resonance energy transfer (FRET) assay previously established for Hsp82 in our lab^4^. The ATP analogs ATPγS or AMP-PNP are used to accumulate the closed state, which results in an increase in FRET efficiency and thus allows us to follow the kinetics of the closing reaction. In this setup, Hsp82 protomers carry fluorophores at cysteine residues in the NTD (C61-ATTO 550) or MD (C385-ATTO 488); for Hsc82 the equivalent positions are C61 and C381 (Supplementary Fig. 3b). Both cysteine variants of Hsc82 support viability in yeast (Supplementary Fig. 3b). Similar to Hsp82, the exchange to cysteines and/or the conjugation of fluorophores in Hsc82 does not strongly affect its ATPase rate (Supplementary Fig. 3b). Mixing of donor- and acceptor-labeled Hsc82 leads to the formation of a FRET-competent hetero-complex that can be tracked by a decrease in the donor fluorescence signal and an increase in the acceptor fluorescence signal (Supplementary Fig. 3b). The subunit exchange rate constants were very similar for both isoforms (Table 3). However, when we compared the closing kinetics upon addition of ATPγS or AMP-PNP, we observed that Hsp82 converts more slowly to the closed conformation than Hsc82, in line with its lower ATPase activity (Table 3). We next tested how co-chaperones affect the closing kinetics. Aha1 and Cpr6 stimulated closure of Hsp82 and of Hsc82, but both co-chaperones had a stronger stimulatory effect on Hsp82 (Fig. 3g and Table 3). Conversely, Sti1 and Cdc37 decelerated the closing kinetics, but had a stronger inhibitory influence on Hsc82 than on Hsp82 (Fig. 3g and Table 3). Strikingly, Sba1/p23 accelerated closing of Hsp82 and decelerated closing of Hsp82. Thus, the overall effect of the co-chaperones on the closing kinetics is to reduce the difference in the closing kinetics of the faster closing Hsc82 and the slower closing Hsp82.

**Table 3:** FRET analysis of the two isoforms

NM-FRET dimer	Hsp82 dimer	Hsc82 dimer	Heterodimer
Subunit exchange rate (*k*_se_ (sec^−1^))	0.034 ± 0.004	0.037 ± 0.006	0.030 ± 0.003
Closing rate (*k*_app_ (min^−1^))	0.190 ± 0.010	0.360 ± 0.000	0.260 ± 0.010
Change with Aha1 (percentage of *k*_app_ without co-chaperone)	868%	616%	
Change with Cpr6 (percentage of *k*_app_ without co-chaperone)	239%	108%	
Change with Cdc37 (percentage of *k*_app_ without co-chaperone)	84%	51%	
Change with Sba1 (percentage of *k*_app_ without co-chaperone)	122%	78%	
Change with Sti1 (percentage of *k*_app_ without co-chaperone)	40%	19%	
Reopening of closed state rate			
Without nucleotide (t½ (min^−1^)	0.564 ± 0.079	0.533 ± 0.085	
With ATP (*k*_app_ (min^−1^)	0.7 ± 0.1	0.7 ± 0.2	
With ATPγS (*k*_app_ (min^−1^)	35.5 ± 3.2	54.2 ± 6.0	
With ATPγS and Aha1 (*k*_app_ (min^−1^)	103.0 ± 2.1	160.0 ± 8.0	
With ATPγS and Sba1 (*k*_app_ (min^−1^)	47.0 ± 1.7	56.0 ± 2.0	
With AMP-PNP (*k*_app_ (min^−1^)	>300	>300	

FRET between the NTD of one protomer and the MD of the other protomers (NM-FRET) was recorded with protomers that had fluorescent dyes attached at C61 or C385 (Hsp82) or C381 (Hsc82). FRET in the absence of nucleotides was used to obtain subunit exchange rates (k_se_) between protomers^4^. FRET in the presence of ATPγS (which stabilizes the closed state) was used to monitor the closing kinetics^4^. FRET chase experiments, which report on the stability of the closed complex, were performed by addition of an excess of unlabeled Hsp90 to a preformed Hsp90 FRET complex (preformed with ATPγS or AMP-PNP)^4^.

### Dimer stability and heterodimer formation

We next explored if the closed states of Hsp82 and Hsc82 differ in their stabilities and performed FRET-chase experiments, in which the disruption of a FRET-complex is initiated by adding an excess of unlabeled Hsp82/Hsc82 and monitored by recording the acceptor fluorescence signal^4^ (Table 3). A fast subunit exchange with a half-life of 0.6–0.7 min^−1^ was observed for both isoforms in the presence of ATP and without nucleotide (Supplementary Fig. 3c). The equilibria of both isoforms were shifted completely towards a stable closed conformation in the presence of AMP-PNP (Supplementary Fig. 3c), in line with what we have previously reported for Hsp82^4^. In contrast, in the presence of ATPγS, Hsc82 displayed a slightly more stable closed conformation compared with Hsp82 as deduced from slower complex disruption. We also observed this in the presence of Aha1 and Sba1 (Fig. 4b and Table 3).

In addition to the closed state, we also explored the open state, i.e., the state where only the CTDs are dimerized. The analysis of Hsp82’s C-terminal dimerization monitored by size-exclusion HPLC had previously revealed a dissociation constant *K*_*d*_ of ≈60 nM^23^. We monitored the subunit exchange in vitro by FRET after mixing of NTD- and MD-labeled dimers (Fig. 4a). Hsp82 and Hsc82 displayed similar exchange rates of ≈0.03 s^−1^, indicating that no differences concerning C-terminal dimerization exist. Moreover, Hsp82 and Hsc82 readily formed heterodimers in vitro with rate constants equal to the homodimers (Table 3). This strongly differs from the human system, where C-terminal heterodimerization is disfavored^31^. The constitutive dimerization of Hsp90 is mediated by a three helix-coil motif in the CTD^20^ (Fig. 4c). As three of the five residues in the CTD that differ between Hsp82 and Hsc82 are a part of this structure (Fig. 1b), we also tested the formation of heterodimers in the cell. To this end, we constructed a yeast strain in which one isoform carries a C-terminal GFP-tag and the other a 6HA-tag. Immunoprecipitation of the GFP-carrying isoform resulted in the co-precipitation of the 6HA-tagged isoform, indicating that Hsp82 and Hsc82 hetero-dimerize in vivo (Fig. 4d). The formation of heterodimers raised the question how the protomers influence each other in the heterodimer. Regarding the ATPase activity and the closing rate, no dominating influence of one protomer on the other was observed because the respective rates of the heterodimers were the average of the rates of the homodimers (Fig. 4e, f and Table 3).

### Interactome analysis of the Hsp90 isoforms

The two most comprehensive genetic screens for yeast Hsp90 functions performed so far suggest that Hsp90 is involved in many processes including precursor metabolism, energy production, and respiration^18,19^. To confirm that Hsp90 is indeed involved in these processes we used stationary yeast cultures. To get isoform-specific information, we expressed either GFP-tagged Hsp82 or Hsc82 as a sole source of Hsp90. The GFP-tags had no significant influence on the growth of the respective strains (Fig. 4g, h). Many interactions of Hsp90 and its clients are transient and/or dependent on ATP, which is rapidly depleted, once cells are lysed^32^. Therefore, we stabilized interacting proteins prior to cell lysis with the cross-linker formalin^33^. We then performed co-immunoprecipitations followed by quantitative mass spectrometric analysis using tandem mass tag (TMT) labeling. Pulldowns were performed with four biological replicates, and equal numbers of matched control samples (expressing the untagged isoforms) were used. We also subjected the input lysates to mass spectrometric analysis in order to monitor the changes due to expression of Hsp82 or Hsc82 and their tagged counterparts. In addition, to monitor the influence of a heat shock, we repeated the experiment with yeast that were additionally exposed to a 30 min heat shock at 42 °C.

In total, we ran 8 TMT 8-plex experiments, four input lysates (2× non-heat shock and 2× heat shock) and four pulldowns (2× non-heat shock and 2× heat shock). Each 8-plex experiment contained two replicates of Hsp82-GFP with the respective Hsp82 control and two replicates of Hsc82-GFP with the respective Hsc82 controls (Supplementary Fig. 6a). We only used proteins which were quantified in all four replicates per condition. The protein identifications are visualized in an UpSetR plot^34^ (Supplementary Fig. 6b).

**Fig. 6:**
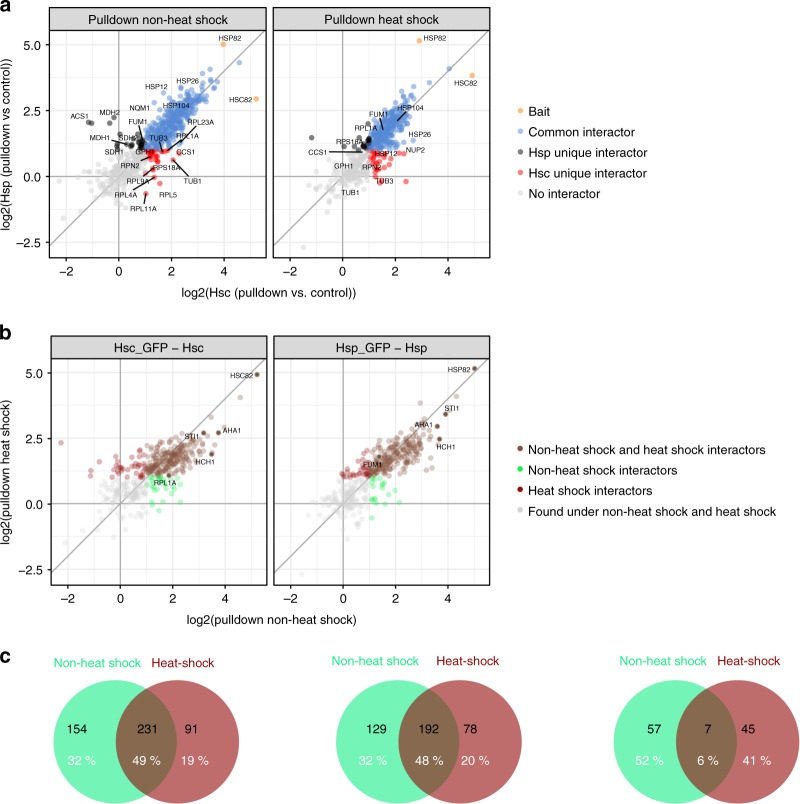
Interactors of the yeast Hsp90 isoforms. **a** Correlation plot of enrichments ratios (pulldown against control) of Hsc-GFP versus Hsp-GFP. Proteins that were significantly enriched (log2 FC ≥ 1, fdr ≤ 0.05) in the pulldowns of both isoforms are categorized as “common interactors”. Proteins that were only significantly enriched (same criteria for enrichment as above) in the pulldown of a single isoform were categorized as Hsp unique or Hsc unique, respectively. **b** Correlation of interactors under non-heat shock and heat shock conditions. Proteins that were only significantly enriched in the non-heat shock sample (termed non-heat shock interactors) or the heat shock sample (heat-shock interactors) and those that were significantly enriched in both samples are displayed in different colors. Criteria for significant enrichment were the same as in Fig. 6a. **c** Venn diagrams displaying the overlap of the interactors described in Fig. 6a, b in numbers

Proteins which were enriched twofold with a false discovery rate smaller 5% (using limma to test for differential abundance^35^) against the corresponding control were called hits (see Volcano plot in Supplementary Fig. 7). For the pulldowns, only hits with positive fold changes were allowed (referred to as interactors). In total we identified ~480 interactors (Fig. 6c, Supplementary Fig. 6c and Supplementary Data 2). We compared them with all yeast Hsp82 and Hsc82 interactors that are currently deposited in the BIOGRID database and found that ~50% of them have not yet been deposited there.

A quantitative comparison of the global proteomes of tagged Hsp82 and Hsc82 strains revealed only minor changes (Supplementary Fig. 7 and Supplementary Data 1), consistent with the lack of a phenotype upon overexpression of a single isoform (Supplementary Fig. 3a). We also found that differences in the global expression in the strains expressing the tagged and the untagged isoforms had no significant effect on the proteins identified in the pulldowns (Supplementary Fig. 7). Leu2 (expressed from the p425GPD plasmid that expresses the tagged protein) and His3 (expressed from the p423GPD plasmid that expressed the untagged protein) were used as controls.

Approximately half of our interactors were significantly enriched in both the non-heat shock experiment and the heat shock experiment (Fig. 6c, Supplementary Fig. 6c and Supplementary Data 2). We categorized the 154 proteins that were exclusively enriched under non-heat shock as “non-heat shock interactors” and the 91 that were exclusively enriched in the heat shocked sample as “heat shock interactors” (Fig. 6b, c). We next classified the interactors according to whether they were “common interactors” or specific for Hsp82 (“Hsp unique interactors”) or Hsc82 (“Hsc unique interactors”) (Fig. 6a). It should be noted that a similar number of proteins was identified with and without heat shock (Supplementary Fig. 6b). Interestingly, we found that some interactors switch their category (from common to unique and vice versa) under non-heat shock and heat shock conditions (Supplementary Data 2). We also found switches between Hsc and Hsp unique interactors (Supplementary Fig. 9a).

### Total interactomes under non-heat shock versus heat shock

We first compared the biophysical properties (mass, isoelectric point (pI) and the overall hydrophobicity (GRAVY index)) of the total of our 476 interactors with the yeast proteome and analyzed if the 154 non-heat shock interactors deviate in their properties form the 91 heat shock interactors. All of our interactors displayed a distribution of molecular masses that was comparable with the yeast proteome, and only a slight trend towards larger molecular masses (>100 kDa) was observed for the heat-shock interactors compared with the non-heat shock interactors (Supplementary Fig. 8a). Intriguingly, our total interactome was strongly enriched in proteins with a pI lower than 7 compared with the total yeast proteome and proteins with a pI between 5 and 6 were overrepresented among the heat shock interactors compared with the non-heat shock interactors (Supplementary Fig. 8a). We also found that hydrophobic proteins were overrepresented in our interactome compared with the yeast proteome. This trend was particularly prominent among the non-heat shock interactors, while the heat shock interactors contained a larger fraction of polar proteins, resembling the total yeast proteome in their distribution (Supplementary Fig. 8a).

To determine if our interactors are enriched in certain protein folds, we next assigned SCOPe folds to the interactors using the SCOPe 2.07 database (Supplementary Data 2 and 3). In total, our interactors belong to seven different SCOPe protein classes (classes a–g) and were enriched in class c proteins, which was also observed for both the non-heat shock and heat shock interactors (Supplementary Fig. 8b). Surprisingly, we found that all enriched class b protein folds were barrels with Greek key topology. Likewise, except for the TIM α/β barrel, all other classes c proteins displayed a three-layer α/β/α topology, suggesting that yeast Hsp90 might have strong affinities for these topologies (Supplementary Data 3). The non-heat shock and the heat shock interactors also showed enrichments of these topologies (Supplementary Data 3).

To analyze the enrichment of biological processes, we used the functional annotation tool provided by the Database for Annotation, Visualization, and Integrated Discovery (DAVID). We found that in total the interactors were enriched in processes linked to translation, precursor biosynthesis, redox homeostasis, and vesicle-mediated transport, in line with what has previously been reported^18,19^. For the heat shock interactors a strong linkage to translational initiation was observed (Supplementary Fig. 8c).

### Unique interactors

To better understand what distinguishes the unique interactors form the total of interactors, we analyzed their folds, biophysical properties, and the biological processes they are involved in. The unique interactors only showed a limited overlap of their folds to the common interactors (Supplementary Fig. 9a). A list of the folds that were exclusively found among the Hsp unique interactors and the folds that were restricted to Hsc unique interactors is given in Supplementary Data 3. The Hsp unique interactors were enriched in proteins with higher pI than the total of interactors or the Hsc unique interactors, respectively, suggesting that Hsp82 might provide specialized support for relatively basic proteins (Supplementary Fig. 9b). The molecular masses and hydropathies were comparable between the Hsp and Hsc unique interactors and similar to the total of interactors (Supplementary Data 2).

Taken as a total, unique interactors are enriched in the numbers of proteins linked to translational initiation (Supplementary Fig. 9c). In fact, we found that 8 out of the 11 initiation factor subunits (73%) that we identified in our experiment are unique interactors (Supplementary Data 2).

## Discussion

In the past decades, important progress in our understanding of Hsp90’s structure, its conformational cycle and interactions with co-chaperones has been achieved. However, isoform differences have remained poorly defined. To address this issue we have performed a comprehensive analysis of the two Hsp90 homologs from *S. cerevisiae*, the constitutively expressed Hsc82 and the stress-inducible Hsp82. We not only investigated whether the isoforms display structural and/or biophysical differences but also tested them for differences in growth and client set under non-heat shock and heat shock conditions.

Although Hsp82 and Hsc82 share the same overall structure, Hsp82 exhibits a higher thermal stability. This is not paralleled by an increase in the mechanical stability of its domains, but rather by an increase in its refolding efficiency, fitting well to Hsp82’s induction under heat shock^8^. Since most of the sequence variations between the isoforms are in the NTD, we took a closer look at the proteins’ ATPases and inhibitor binding. It has previously been reported that the expression of human Hsp90β makes cells more sensitive toward the inhibitor RD than human Hps90α^26^. It is, however, difficult to relate this finding to yeast, because the yeast isoforms resemble Hsp90β and Hps90α to a similar degree. Interestingly, both the ATPases and the in vivo sensitivity towards RD differed between the yeast isoforms, despite similar affinities for the respective ligands. NMR revealed that in the apostate, several residues in the NTDs differ in their structural environment. The regions affected comprise the ATP-binding pocket, namely loops 81–85 and 139–145, helix α3 and the C-terminal part of α2. These regions affect interactions with active site ligands^29^. In the presence of RD, several residues that are involved in key interactions with the inhibitor exhibit distinct chemical shifts in the two isoforms. These differences may indicate different conformations and/or changes in internal motion, which contribute to the allosteric communication within the protein. In contrast, the binding of ATP did not differ significantly between Hsp82 and Hsc82. Instead, here, we detected effects for specific residues involved in interdomain contacts, especially in the highly dynamic strand β1 and helix α1. Together, these observations suggest that the changes in inhibitor sensitivity and ATPase activity of the full-length proteins originate from a different conformational and/or dynamic response of the NTD to the binding of ligands, which affect allosteric communication and ultimately the overall structural transitions of the dimer.

Our FRET experiments support the view that the isoforms display differences in their structural transitions. Hsc82 converts two times faster to the closed state than Hsp82 and stays ~1.5 times longer in the closed state than Hsp82, which is in agreement with the higher ATPase of Hsc82. The observed small increase in the stability of the charged linker of Hsc82 could contribute to this. Despite similar binding affinities also co-chaperones affect the isoforms differently. Overall, the slower closing of Hsp82 is accelerated more (by accelerating co-chaperones) and decelerated less (by decelerating co-chaperones) than Hsc82. Therefore, the closing kinetics of the isoforms are more similar in the presence of co-chaperones than without them. p23/Sba1 has the most extreme effect in this regard: It accelerates closing Hsp82 and decelerates closing of Hsc82. A differential impact of p23/Sba1 on the isoforms is also supported by our NMR analysis, which shows that the binding sites of p23/Sba1 in the NTDs involve residues with the largest differences between the isoforms (Supplementary Fig. 10).

In principle, the differences between the isoforms described here could be further modulated by the numerous posttranslational modifications (PTMs) of Hsp90^36,37^. However, since none of the known PTM target residues differ between the isoforms, we assume that PTMs would equally affect the two isoforms. Whether the effects we observe for the yeast Hsp90 isoforms are conserved remains to be seen. The enzymatic activities of the human Hsp90 isoforms have been compared with some extent^31^. The picture emerging seems to be different from yeast because the ATPase activities of both human isoforms are highly similar. It should also be considered that the number of sequence changes is much larger for the human isoforms than between the yeast isoforms. There are 91 sequence changes in the human system compared with 16 sequence changes in the yeast system. Thus, overall, the human MD and CTD might contribute more to isoform-specific differences, (e.g., to differences in the human isoforms’ affinities for co-chaperones^38^) than what we observe in this study for yeast.

Our interactome analysis reveals that most clients that we find are pulled down with both isoforms. These clients are related to many processes that Hsp90 has been previously described to be involved in^18^, in particular translation, redox homeostasis and vesicle-mediated transport. However, our data also show that yeast Hsp90 seems to have a prominent role in chaperoning enzymes that are linked to nutrient deprivation (TCA cycle and gluconeogenesis), suggesting that next to heat shock, yeast Hsp90 might also have a prominent role in combating stationary phase stress (or starvation). In terms of function, we do not find many differences under non-heat shock and heat shock, except that under heat shock yeast Hsp90 seems to become particularly important for translation.

Our data also give some insights into the biophysical traits and structures that yeast Hsp90 binds to. The clients that we find are enriched in proteins with a pI lower than 7. These proteins might have a stronger tendency to unfold than proteins with a high pI as their pI is similar to yeast’s intracellular pH. This notion is supported by our finding that clients with a pI between 5 and 6 are underrepresented among the non-heat shock interactors and overrepresented during heat shock interactors, where the intracellular pH drops in this range. We also find that yeast Hsp90 clients are enriched in relatively hydrophobic proteins. This is expected as these proteins are more prone to aggregate. Interestingly, more polar proteins are found in the interactome under heat shock, indicating that they then might also become prone to aggregation.

Most clients that we find are class c proteins. Intriguingly, all class c proteins that we find (except for the TIM barrel) display a three-layer α/β/α topology. Likewise, all class b clients are barrels with Greek key topologies, suggesting that yeast Hsp90 has a pronounced tendency to bind proteins with these two topologies (in the many flavors in which they come). No significant alterations in the folds or topologies are found between non-heat shock and heat shock interactors. Many of the α/β folds that we find have also been identified as GroEL clients^39^. As some obligate GroEL clients cannot be processed by the chaperone CCT/TRiC when expressed in eukaryotes, it was previously proposed that a different eukaryotic chaperone might have taken over this task^39^. Based on our results we like to think that Hsp90 is this factor.

It has been suggested previously that Hsp90 might have a preference for ligand-binding proteins^16,40^. The folds we find support this view. However, most ATP-binding proteins identified here are enzymes involved in the synthesis of nucleotides, porphyrins, and carbohydrates, rather than kinases. This differs strongly from mammals, where 60% of all protein kinases interact with Hsp90.

Our data also reveal a small number of proteins that are only enriched with a specific isoform (unique interactors). Due to their limited number, we cannot tell if they are enriched in certain folds/topologies and we also performed the GO term analysis with all of them rather than comparing the distinct groups (Hsp unique interactors versus Hsc unique interactors). The unique interactors show a stronger linkage to translational initiation compared with the total sum of interactors. We also find that Hsp unique interactors are enriched in proteins with a higher pH. Since basic proteins might not fit the general yeast Hsp90 client scheme, this could indicate that one function of Hsp82 is to provide specialized support to proteins with this feature under heat shock.

In summary, our study reveals a precise tuning of the cognate and the stress-induced isoform. For shifting yeast Hsp90’s properties only a few mutations are required, but ultimately these mutations allow cells that express Hsp82 to grow better than cells lacking this isoform under heat stress and potentially other proteotoxic conditions.

## Methods

### Cloning

Hsp82 and Hsc82 were amplified by PCR and cloned via the restriction sites BamHI and XhoI into a modified pET28 vector (Invitrogen, Karlsruhe, Germany) containing a 6xHisSUMO-tag sequence. This allows the cleavage of the His-tag using the SUMO protease. All Hsp90 chimeras used in the in vivo assays were generated using the sequence- and ligation-independent cloning method^41^. The Hsp90 point mutants and domain swap variants were generated using Quick Change (Stratagene, La Jolla, USA) site directed mutagenesis with p423GPD containing wild-type yeast Hsp82 as a template vector.

### Protein expression and purification

The proteins were expressed in the *E. coli* strain BL21 (DE3) RIL (Stratagene, La Jolla, USA) and purified slightly modified to remove the precursor tag according to standard protocols^31^ and stored in 40 mM HEPES pH 7.5, 150 mM KCl, 5 mM MgCl_2_ (standard buffer) at −80 °C until usage. For the NMR samples, minimal media containing 95% D_2_O and supplemented with U-^13^C glucose (Cambridge Isotope Laboratories, Tewksbury, USA) and ^15^NH_4_Cl (Cortecnet, Paris, France) were used for growing the cells and expressing the protein. Briefly, cells were lysed using a cell disruption system (Constant Systems, Warwick, UK) in a buffer composed of 50 mM sodium phosphate pH 7.8 and 300 mM NaCl. For separation of protein-containing lysate and cell debris the sample was centrifuged (18,000 g; 45 min; 8 °C). The Hsp90 isoforms were first purified using Ni-NTA affinity chromatography (GE Bioscience, Munich, Germany) and were eluted with a gradient of buffer containing 50 mM sodium phosphate pH 7.8, 300 mM NaCl and 300 mM imidazole. The proteins were further purified by ResourceQ anion exchange (GE Bioscience, Munich, Germany) with a buffer system composed of a low salt buffer (20 mM Hepes, pH 7.5 and 50 mM KCl) and a high salt buffer (20 mM Hepes, pH 7.5 and 1 M KCl). Then the 6×His-tag was cleaved off by incubation with SUMO protease overnight at 4 °C. The tag and the protease were removed again by using a second Ni-NTA affinity chromatography in the buffer system described. Finally, Superdex200 pregrade size exclusion chromatography (GE Bioscience, Munich, Germany) in standard buffer (40 mM Hepes pH 7.5, 150 mM KCl, 5 mM MgCl_2_) was used as a last purification step. His-tagged Hsp90 and the NTDs of Hsp90 were purified with chromatography steps and buffers similar to the tagged isoforms, except that the tag was not removed. For the purification of co-chaperons NI-NTA chromatography and size exclusion chromatography. Buffer systems were identical to those used for the purification of the Hsp90 isoforms. The purity of the samples was verified by SDS-PAGE and mass spectrometry.

### ATPase activity

ATPase assays were performed using an ATP-regenerating system, in which the rate of absorbance decrease of NADH at 340 nm is used as a read-out for ATP consumption^23,31^. Assays were measured with an Hsp90 concentration of 3 μM in standard buffer (40 mM HEPES, pH 7.5, 150 mM KCl, 5 mM MgCl_2_) supplemented with 2 mM ATP (Roche, Mannheim, Germany) at 30 °C. The Hsp90-specific ATPase activity was inhibited by adding 50 μM of the inhibitor RD (Sigma, St. Louis, USA) and the remaining activity was subtracted as background. The assays were evaluated using Origin software (OriginLab Corporation, Northhampton, USA). Results are the mean of at least three independent experiments. Error bars indicate standard errors. The stimulation of the ATPase activity by co-chaperones was measured under comparable conditions except for using 1 µM of Hsp90 and low salt buffer (40 mM HEPES, pH 7.5, 20 mM KCl, 5 mM MgCl_2_).

### CD spectroscopy

Far-UV CD spectroscopy was performed from 190 to 260 nm. The protein samples were dialyzed against 50 mM sodium phosphate buffer, pH 7.5. The protein concentration was 0.2 mg/ml. The measurements were performed at 20 °C in a Jasco J170 spectropolarimeter (Jasco, Groß-Umstadt, Germany). All CD-spectra were corrected by subtraction of the buffer spectrum and the data were expressed as mean residue weight ellipticity.

### Protein labeling

Labeling of the Hsp90 cysteine variants was performed with ATTO-488 maleimide and ATTO- 550 maleimide (ATTO-TEC, Siegen, Germany) in PBS buffer pH 7.4 using a twofold molar excess of reactive dye compared with the protein and incubation for 2 h at RT. Free labels were removed via a Superdex 75 column (Pharmacia Biotech) equilibrated in PBS buffer pH 7.4. The labeled protein was concentrated and the degree of labeling was determined by UV-VIS spectroscopy.

### FRET analysis

All FRET experiments were performed to analyze the conformational rearrangements after nucleotide binding^4^. Hsp90 heterodimers (400 nM) were formed by mixing an equal amount of donor-labeled and acceptor-labeled Hsp90 in standard buffer. For the experiments in the presence of co-chaperones, the respective co-chaperone (4 µM) was added prior to recording the conformational kinetics and incubated for 15 min at 30 °C. The experiment was started by addition of 2 mM nucleotide (ATP, ATPγS, AMP-PNP from Roche, Mannheim, Germany) and the increase of fluorescence intensity was recorded at 30 °C using a Fluoromax 2 fluorescence spectrophotometer (Horiba Jobin Yvon, München, Germany) at 575 nm after excitement at 490 nm. The apparent rate constants *k*_app_ of the conformational changes were determined by fitting the data to a monoexponential function using Origin software (OriginLab Corporation, Northhampton, USA).

### Subunit exchange

The subunit exchange rates of homo- and hetero-dimers were tracked in FRET chase experiments^4^. Homo- and hetero-dimers were performed as described for FRET analysis experiments. The subunit exchange was monitored by addition of a tenfold excess of unlabeled Hsp90 and the decay of fluorescence intensity was recorded using a Fluoromax-2 fluorescence spectrophotometer (Horiba Jobin Yvon, München, Germany) at 575 nm after excitement at 490 nm at 30 °C. For experiments in the presence of different nucleotides (ATP, ATPγS, (Roche, Mannheim, Germany)), Hsp90 variants were preequilibrated for 30 min in the presence of 2 mM of the respective nucleotide to allow the formation of the closed state. For the experiments in presence of co-chaperones, the respective co-chaperone (4 µM) was added prior to recording the subunit exchange and incubated for 15 min at 30 °C. The apparent half-life of the reaction was determined by fitting the data using the function for exponential decay in the Origin software (OriginLab Corporation, Northhampton, USA).

### NMR spectroscopy

NTDs of Hsp82 and Hsc82 uniformly labeled with ^15^N/^13^C/^2^H (~95% deuterium enrichment) were prepared for backbone assignment experiments. Samples were concentrated to 500 µM in 20 mM sodium phosphate pH 6.5, 100 mM NaCl, 2 mM EDTA, 1 mM DTT, 0.02% NaN_3_, and 5% D_2_O. 5 mM MgCl_2_ and ATP, and 2 mM RD in DMSO-d6 were added in order to obtain the corresponding bound forms. To minimize the effects of the intrinsic ATP hydrolysis in the 3D experiments, nonuniform sampling was used to shorten the measurement time, together with an ATP regenerating system^42^. For the backbone assignment, a series of triple resonance HNCA, HNCACB, HN(CO)CA, HNCO, and HN(CA)CO experiments^43^ were performed for apo, ATP- and RD-bound Hsc82. Additional ^15^N-edited NOESY-HSQC experiments were performed in the case of apo proteins (mixing time of 120 ms). All experiments were performed at 25 °C in a Bruker Avance III 800 MHz spectrometer equipped with a cryogenically cooled probe, with the exception of the NOESY experiments, which were carried out at 600 MHz. Spectra were processed using Bruker Topspin 3.5 software (Bruker, Billerica, USA) and analyzed using CcpNmr Analysis^44^. CSP values were calculated according to the following formula:

1 $$\Delta \delta _{N,H}\left( {ppm} \right) = \sqrt {\Delta \delta _H^2 + \left( {\alpha \cdot \Delta \delta _N} \right)^2},$$

where *α* is a scaling factor calculated from the ratio between the ^1^H and ^15^N chemical shift ranges (*α* = 0.1689).

### Single molecule force spectroscopy using optical tweezers

Optical tweezers measurements were performed using a custom-built experimental setup^45^. Full-length Hsp82 and Hsc82 and the NTDs were expressed and purified as described above. For protein-DNA coupling, cysteine residues were reduced with 10 mM TCEP for 15 min at 20 °C followed by TCEP and buffer exchange to 40 mM HEPES buffer containing 150 mM KCl and 10 mM MgCl_2_ pH 7.4 using a Superdex 200 column (GE Healthcare, USA). Protein and DNA oligonucleotides with a 3′-maleimide modification were incubated for 1 hour at 20 °C 40 mM HEPES buffer containing 150 mM KCl and 10 mM MgCl_2_ pH 7.4. Unreacted DNA oligonucleotides were then removed, again using a Superdex 200 column. For measurements samples were prepared by incubating the protein–oligonucleotide construct with 370-nm-long dsDNA handles containing either biotin or digoxigenin modifications at 5′ and an overhang complementary to the protein-coupled oligonucleotide at 3′. For optical tweezers measurements, these were incubated with streptavidin-coated 1 µm silica beads (Bangs laboratories, USA) in measurement buffer (40 mM HEPES, 150 mM KCl, 10 mM MgCl2, pH 7.4) for 20 min before being diluted in measurement buffer and mixed with antidigoxigenin-coated 1 µm silica beads (custom functionalization in-house, beads from Bangs laboratories, USA). An oxygen scavenger system was used, to reduce photodamage. This comprises (concentrations in final volume): glucose 0.33% (Sigma, Germany), 13 U/ml glucose oxidase (Sigma, Germany), 8500 U/ml catalase (Calbiochem, Germany). Where RD was used, this was added into the final solution to a concentration of 10 µM. The constant velocity optical tweezers measurements and data evaluation were performed as reported in^3,12^. Briefly, force is applied to the protein through the formation of a dumbbell between two optically trapped beads. This dumbbell consists of the protein, the two dsDNA handles and one streptavidin-coated bead and one antidigoxigenin-coated bead. By moving the position of the focus of the laser beam which is holding one of the beads, the bead separation is increased and a force is applied to the protein-DNA construct. A typical force-extension trace is obtained by moving the beads apart at a constant velocity to observe protein unfolding, and then moving them back together at a constant velocity to observe relaxation of the protein-DNA construct resulting in protein refolding. The position of the laser beam is controlled by a piezo mirror (Mad City Labs, USA). The obtained force-extension traces can be fitted with a worm-like chain model to obtain the change in contour length for each unfolding event. This change in length for each unfolding event is then compared with the expected changes in length for different parts of the protein structure, based on the number of amino acids comprising each domain, and thus assigned. Once a native signature is established, the refolding capability of the protein under the measured conditions is assessed by measuring one unfolding trace and then counting how many native events appear in subsequent unfolding traces for the same molecule.

### Analytical ultracentrifugation

Interaction between the Hsp90 isoforms and co-chaperones were analyzed using AUC with ATTO 488 labeled proteins in a Beckman ProteomeLab XL-A (Beckman) equipped with a fluorescence detection system (Aviv Biomedica). Sedimentation-velocity experiments were performed with ATTO 488-labeled proteins and unlabeled proteins in 40 mM Hepes, pH 7.5, 150 mM KCl, 5 mM MgCl_2_) and the indicated nucleotides at 42,000 rpm. A Ti-50 rotor (Beckman) was used at 20 °C. To determine the sizes of complexes, the raw data were converted to *dc*/*dt* profiles by subtraction of nearby scans and conversion of the difference into *dc*/*dt* plots. *dc*/*dt* profiles were analyzed to determine the *s* values and the areas of the corresponding peaks.

### FOA plasmid shuffling assay

The in vivo functionality of the different Hsp90 variants was tested using a plasmid shuffling approach and strain that has been constructed by the Lindquist lab^46,47^. This yeast strain is deficient in genomic *HSP82* and *HSC82* and contains a plasmid with a URA3 selection marker coding for Hsp82 to rescue lethality. The URA3 selection marker enables a selection for cells that have lost the wild-type Hsp82 plasmid in a medium supplemented with 5-FOA. Hsp90 wild type and the mutant-variants were constitutively expressed from a 2 micron high-copy number plasmid under the control of a constitutive glyceraldehyde-3-phosphate dehydrogenase gene (GPD) promotor (p423GPD vector). The cells surviving the shuffling were tested for loss of the URA3-plasmid by growth in media lacking uracil.

### Growth and RD sensitivity assays

To examine growth of yeast expressing either Hsp82 or Hsc82 (and their tagged counterparts), strains expressing p423GFP-Hsp82, p423GFP-Hsc82, p425-Hsp-GFP, and p425-Hsc-GFP were constructed using the shuffling approach described above. A total of 5 ml of rich (YPD) medium were inoculated with the respective strains and cells were grown to mid-log phase at 30 or 42 °C, respectively. Cells were then diluted back to an OD_600_ of 0.1 and grown in Sarstedt 96-well plates for 16 h in a TECAN infinite 200Pro or PHERAstar plus microplate reader. The temperature was set to 30 or 42 °C, respectively, shaking was performed at 600 rpm and growth was recorded at a wavelength of 600 nm. To allow comparison of the data that were obtained with the two different instruments used (TECAN infinite 200Pro or PHERAstar plus microplate reader), the OD reached at the end of the incubation time was set to 1. Biological triplicates of each strain were used to calculate standard deviations.

For the RD assay yeast containing p423GPD plasmids expressing the Hsp90 variants were grown to stationary phase overnight in SC medium lacking histidine. The cells were then diluted back to an OD_600_ of 0.1 and grown at 30 °C for 20 h in SC medium supplemented with RD (Sigma, St. Louis, USA). The optical density (OD_600_) was used as read-out and standard deviations were calculated based on biological triplicates.

### Co-immunoprecipitations and western blot

Hetero-dimer formation of the Hsp90 isoforms was assessed in yeast strains in which one Hsp90 isoform had a C-terminal GFP-tag and the other isoform had a C-terminal 6HA-tag. Tagging of the endogenous *HSP90* isoforms was performed according to the protocol provided by Janke et al.^45^. Cells were lysed in 50 mM Tris, pH 8, 20 mM NaCl and 0.15% NP40 and protease inhibitor Mix G (Serva). Co-immunoprecipitations were performed by incubating cleared lysates with GFP-Trap_A agarose (Chromotek) or Anti-HA agarose (Sigma) for 30 min. Beads were washes four times with lysis buffer and proteins were eluted by boiling the agarose for 5 min in 4× SDS buffer (NUPAGE). The following primary antibodies were used to probe the membrane: antiyeast Hsp90 polyclonal antibody (Pineda Antibody Service^48^ at a concentration of 1:30,000) anti-PGK1 monoclonal antibody (Novex, cat. no. 459250 at a concentration of 1:15,000), anti-HA monoclonal antibody (Sigma, product number H9658, at a concentration of 1:5000) and anti-GFP antibody (Roche, cat. no. 11814460001, at a concentration of 1:1000). The secondary antibodies were anti-mouse and anti-rabbit IgG-peroxidase conjugated antibodies (Sigma-Aldrich, cat. nos. A9044 and A0545, respectively, both used at a concentration of 1:20,000). Image acquisition was performed with an ImageQuant LAS4000 (GE Healthcare).

### Interactome analysis

To analyze the interactome of the Hsp90 isoforms, plasmids encoding C-terminal GFP-tagged Hsc82 or Hsp82 (p425GPD-Hsc82-GFP and p425GPD-Hsp82-GFP) and the non-tagged isoform versions (p423GPD-Hsc82 and p423GPD-Hsp82) were introduced by plasmid shuffling^49^ into a yeast strain in which both genetic copies of Hsp90 were deleted. Overnight cultures of each strain were grown in YPD and an OD of 200 was used for each experiment. For the heat shock experiment, the respective cultures were exposed to 42 °C for 30 min in a water bath (after overnight growth). Co-immunoprecipitations were performed as described above except that prior to cell lysis interactions were stabilized by incubating yeast for 10 min in 1% formalin minutes followed by quenching with 0.5 M glycine for 10 min. Duplicates of the samples were analyzed simultaneously in one LC-MS/MS run (i.e., in an 8-plex TMT experiment) and in total two of these TMT 8-plex experiments were performed. Only proteins that were identified in both runs and at a FDR of 5% were further analyzed. Significant enrichment was defined as an enrichment of twofold or more. Proteins that were significantly enriched in the pulldown of the tagged isoform compared with the untagged isoform were referred to as interactors.

### Thermal shift assay

The thermal shift assay (TSA) was performed with a real time PCR-cycler (Agilent Technologies Stratagene Mx3000P). A total of 5 µg of each Hsp90 variant was incubated in 20 µl of buffer (40 mM Hepes pH 7.5, 150 mM KCl, and 5 mM MgCl_2_) in the presence of SYPRO Orange (5×). The change of SYPRO Orange fluorescence at 590 nm after excitation at 475 nm was monitored with temperature steps of 1 °C per minute. Melting temperatures were calculated by using the second derivative. Measurements were performed in triplicates.

### Isothermal titration calorimetry

To determine the binding affinity of the Hsp90 NTDs to ATP and RD ITC was applied. Prior to the measurements the Hsp90 NTDs were dialyzed against ITC buffer (40 mM HEPES, pH 7.5, 150 mM KCl, 5 mM MgCl_2_). The same buffer was used to prepare the ligand stock solution. The concentration of the Hsp90 NTD Stock solution was 10 µM and the ATP and RD stock concentrations were 100 µM. The measurements were performed with a MicroCal PEAQ-ITC calorimeter (Malvern Instruments Limited, UK). All ITC measurements were performed at 25 °C. Data analysis was carried out with the MicroCal user software.

### Sample preparation for MS experiments

For the mass spectrometric comparison of protein expression 20 µg of cell lysates were used. For analysis of the interactomes, the total eluates obtained after boiling of the beads in the co-immunoprecipitation experiments were used. In order to provide a more robust quantification and thus statistical analysis all pull-down experiments were analyzed in a combined TMT experiment. Hence, the identification of Hsc82 or Hsp82, respectively, in the “knock-out” cell line can be considered an artifact of the underlying experimental set-up. Since, the absence of either gen has been confirmed on a genetic level. A tryptic digest using a modified version of the Single-Pot Solid-Phase-enhanced Sample Preparation (SP3) protocol^50,51^ was performed. Here, lysates were added to Sera-Mag Beads (Thermo Scientific) in 10 µl 15% formic acid and 30 µl of ethanol. Binding of proteins was achieved by shaking for 15 min at room temperature. Beads were washed four times with 200 µl of 70% ethanol. Proteins were digested with 0.4 µg of sequencing grade modified trypsin (Promega) in 40 µl HEPES/NaOH, pH 8.4 in the presence of 1.25 mM TCEP and 5 mM chloroacetamide (Sigma-Aldrich,) overnight at room temperature. Beads were separated, washed with 10 µl of an aqueous solution of 2% DMSO and the combined eluates were dried down. Peptides were reconstituted in 10 µl of H_2_O and reacted with 80 µg of TMT10plex (Thermo Scientific) label reagent dissolved in 4 µl of acetonitrile for 1 h at room temperature. Excess TMT reagent was quenched by the addition of 4 µl of an aqueous solution of 5% hydroxylamine (Sigma). Peptides were mixed to achieve a 1:1 ratio across all TMT-channels. Mixed peptides were subjected to a reverse phase clean-up step (OASIS HLB 96-well µElution Plate, Waters) and analyzed by LC-MS/MS on a Q Exactive Plus (Thermo Scientific)^52^. For the mass spectrometric comparison of total proteomes, 20 µg of cell lysates were subjected to an in-solution tryptic digest as described above for the analysis of interactomes. However, here, TMT-labeled and mixed peptides were subjected to an offline high pH fractionation^50^. The resulting 12 fractions were, analog to the analysis of the interactomes, analyzed by LC-MS/MS.

### LC-MS/MS measurement

Peptides were separated using an UltiMate 3000 RSLC nano LC system (Thermo Fisher Scientific) equipped with a trapping cartridge (Precolumn C18 PepMap 100, 5 µm, 300 µm i.d. × 5 mm, 100 Å) and an analytical column (Waters nanoEase HSS C18 T3, 75 µm × 25 cm, 1.8 µm, 100 Å). Solvent A was 0.1% formic acid in LC-MS grade water and solvent B was 0.1% formic acid in LC-MS grade acetonitrile. After the trapping step (30 µL/min of solvent A for 3 min), elution was performed with a constant flow of 0.3 µL/min and 120 min of analysis time (with a 2–28%B elution, followed by an increase to 40%B, and reequilibration to initial conditions). The LC system was directly coupled to a Q Exactive Plus mass spectrometer (Thermo Fisher Scientific) using a Nanospray-Flex ion source and a Pico-Tip Emitter 360 µm OD × 20 µm ID; 10 µm tip (New Objective). The mass spectrometer was operated in positive ion mode with a spray voltage of 2.3 kV and capillary temperature of 320 °C. Full scan MS spectra with a mass range of 375–1200 m/z were acquired in profile mode using a resolution of 70,000 (maximum fill time of 30 ms or a maximum of 3e6 ions (automatic gain control, AGC)). Fragmentation was triggered for the top 10 peaks with charge 2–4 on the MS scan (data-dependent acquisition) and precursors were isolated with a quadrupole isolation width of 1 m/z. The dynamic exclusion window was set to 30 s (normalized collision energy was 32), and MS/MS spectra were acquired in profile mode with a resolution of 35,000 (maximum fill time of 120 ms or an AGC target of 2e5 ions).

### Protein identification and quantification

Raw mass spectrometry files were processed with IsobarQuant^53^, peptide and protein identification were performed with Mascot 2.4 (Matrix Science) against the *S. cerevisiae* Uniprot FASTA (Proteome ID: UP000002311), modified to include known contaminants and the reversed protein sequences (search parameters: trypsin; missed cleavages 3; peptide tolerance 10 ppm; MS/MS tolerance 0.02 Da; fixed modifications were carbamidomethyl on cysteines and TMT10plex on lysine; variable modifications included acetylation on protein N-terminus, oxidation of methionine and TMT10plex on peptide N-termini). The FDR for protein identification was 1%.

### Data analysis

The R programming language (https://www.r-project.org) was used to process the raw output data of IsobarQuant. As a quality filter, only proteins that were quantified with at least two unique peptide matches were used for further analysis. Furthermore, only proteins that were quantified in all replicates were kept for downstream analysis. First, potential batch effects were removed using a function from the limma package^35^. Second, batch cleaned data were normalized with variance stabilization using the vsn package^54^. Testing for differential abundance using a moderated *t*-test was performed with limma again. The replicate information was included as a covariate into the linear model. Proteins were classified as hits/interactors with a fold-change of two and a false discovery rate below 5%. For the pulldown samples, only positive fold-changes were allowed. UpSetR-plots were created using the UpsetR R package^34^. GO direct terms (in the category biological processes) were assigned using the information from the DAVID bioinformatics resource. An EASE score of 0.1 and fold enrichment >3 over the yeast proteome were used as thresholds. Only GO categories that comprise at least 2% of the input data were shown. Venn diagrams were created with the help of an online tool (http://bioinfogp.cnb.csic.es/tools/venny/). SCOPe folds were assigned to the interactors by performing a BLAST-based search with an online tool (http://phosphatome.net/2.0/blast-scop/) and an *e*-value of 0.001 as a similarity threshold for fold assignment. To assign SCOPe folds to the total proteome of the yeast strain S288C (downloaded from the UniProt website) the search tool provided on the supfam.org website was used.

### Reporting summary

Further information on research design is available in the Nature Research Reporting Summary linked to this article.

## Supplementary information

Supplementary Information

Description of Additional Supplementary Files

Supplementary Data 1

Supplementary Data 2

Supplementary Data 3

Reporting Summary

Source Data

## Data Availability

Data are available via ProteomeXchange with the identifier PXD013955. The source data underlying the Figs. 4, 5d, 6a, b and Supplementary Figs. 3a, 6b, c, 7 are provided as a Source Data file. Other data are available from the corresponding author upon reasonable request.
