# Supplementary material for: The Hsp90 isoforms from S. cerevisiae differ in structure, function and client range
Source: Nat Commun. 2019 Aug 9;10:3626. doi: 10.1038/s41467-019-11518-w (PMC6689086; doi:10.1038/s41467-019-11518-w)
Supplement: Supplementary file 1 — Supplementary Information [file 41467_2019_11518_MOESM1_ESM.pdf]

Supplementary Information

The Hsp90 isoforms from *S. cerevisiae* differ in structure, function and client range

Hannah Girstmair, Franziska Tippel, *et al*.

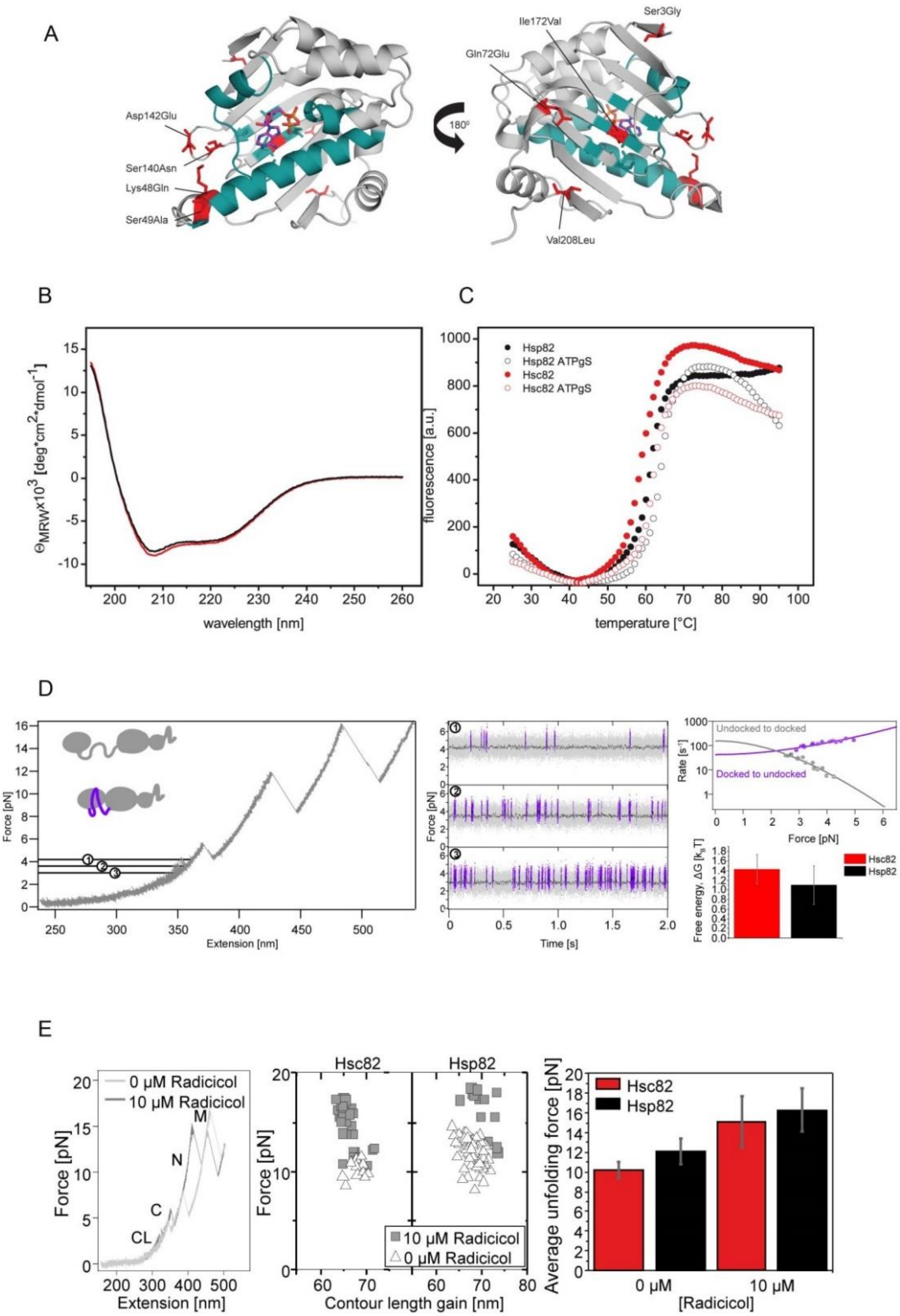

A
Asp142Glu
Ser140Asn
Lys48Gln
Ser49Ala
180°
Ile172Val
Ser3Gly
Gln72Glu
Val208Leu
B
ΘMRWx10^3 [deg*cm^2*dmol^-1]
wavelength [nm]
C
Hsp82
Hsp82 ATPgS
Hsc82
Hsc82 ATPgS
fluorescence [a.u.]
temperature [°C]
D
Force [pN]
Extension [nm]
Time [s]
Undocked to docked
Docked to undocked
Rate [s^-1]
Free energy, ΔG [kBT]
Hsc82
Hsp82
E
0 μM Radicicol
10 μM Radicicol
M
N
C
CL
Force [pN]
Extension [nm]
Hsc82
Hsp82
10 μM Radicicol
0 μM Radicicol
Contour length gain [nm]
Average unfolding force [pN]
0 μM
10 μM
[Radicicol]

Supplementary Fig. 1: Structure and stability of the isoforms. A: Cartoon showing the NTD of Hsp82 with sequence differences between Hsp82 and Hsc82 highlighted (PDB entry 1AM1, [1]). Residues forming the ATP-binding pocket are colored in teal, while changed residues are shown as red sticks. **B:** Far-UV circular dichroism analysis of the two Hsp90 isoforms. **C:** Thermal shift assay with the two Hsp90 isoforms. **D:** The charged linker of Hsc82 compared with that of Hsp82. **Left panel:** A single unfolding trace of Hsc82 pulled at 20nm/s, showing the flipping of the charged linker followed by the unfolding of the CTD, NTD and MD. The inset shows a schematic illustration of the undocked (grey) and docked (purple) states of the charged linker**. Centre**: Performing equilibrium measurements at a constant distance at points (1), (2) and (3) illustrated on the left-hand side, shows the impact of increasing bead separation on the measured forces and kinetics of the docked (purple) and undocked (grey) states of the charged linker. At an increased bead separation, a higher force is applied across the molecule, and the charged linker is less likely to be found in the docked state. This is clearly illustrated on the right-hand side**. Upper right panel:** Transition rates between states vs. force for a single molecule of Hsc82. As force increases, the rate of going from the undocked state to the docked state decreases. **Lower right panel**: A comparison of the calculated stabilization free energy values for the charged linkers of Hsp82 (1.1 k_BT ± 0.4, measured from 34 molecules, error is SD between measurements) and of Hsc82 (1.43 k_BT ± 0.3, measured from 5 molecules, error is SD between measurements). **E:** The impact of RD on the NTD stabilities of Hsp82 and Hsc82. **Left panel:** A single unfolding trace of Hsc82 pulled at 20nm/s without (light grey) and with (dark grey) 10 µM of RD. The flipping of the charged linker (CL) and unfolding of the CTD (C), NTD (N) and MD (M) are annotated. The NTD is significantly stabilized by the presence of RD. **Centre:** Scatter plots of unfolding force vs. contour length gain at a 20nm/s pulling velocity for the NTDs of Hsp82 (left) and Hsc82 (right), showing unfolding events without RD (white triangles) and with 10 µM of RD (dark grey squares). **Right panel:** Average unfolding forces with and without RD from the scatter plot in the central panel, illustrating the impact that RD has in stabilizing the NTDs of both isoforms.

A

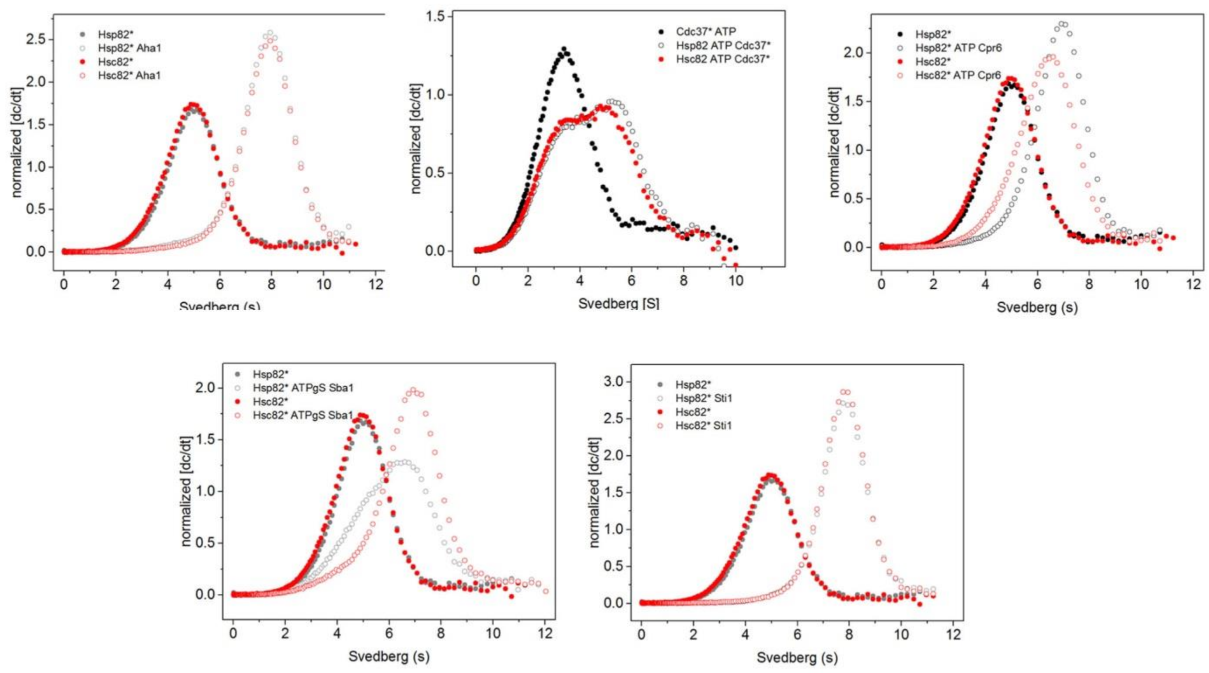

Supplementary Fig. 2: **Chaperone binding and growth of the isoforms**. **A:** Complex formation between the Hsp90 isoforms and co-chaperones determined by analytical ultracentrifugation. Normalized sedimentation profiles (dc/dt) of the Hsp90 isoforms in the presence of: Aha1, Cdc37/ATP, Cpr6/ATP, p23/Sba1/ATPγS and Sti1. All experiments except the one with Cdc37 were performed with Hsp90 isoforms that were labelled with ATTO 488. The Cdc37 experiment was performed with Cdc37 labelled with ATTO 488, since no binding to Cdc37 was detected when labelled Hsp90 isoforms were used.

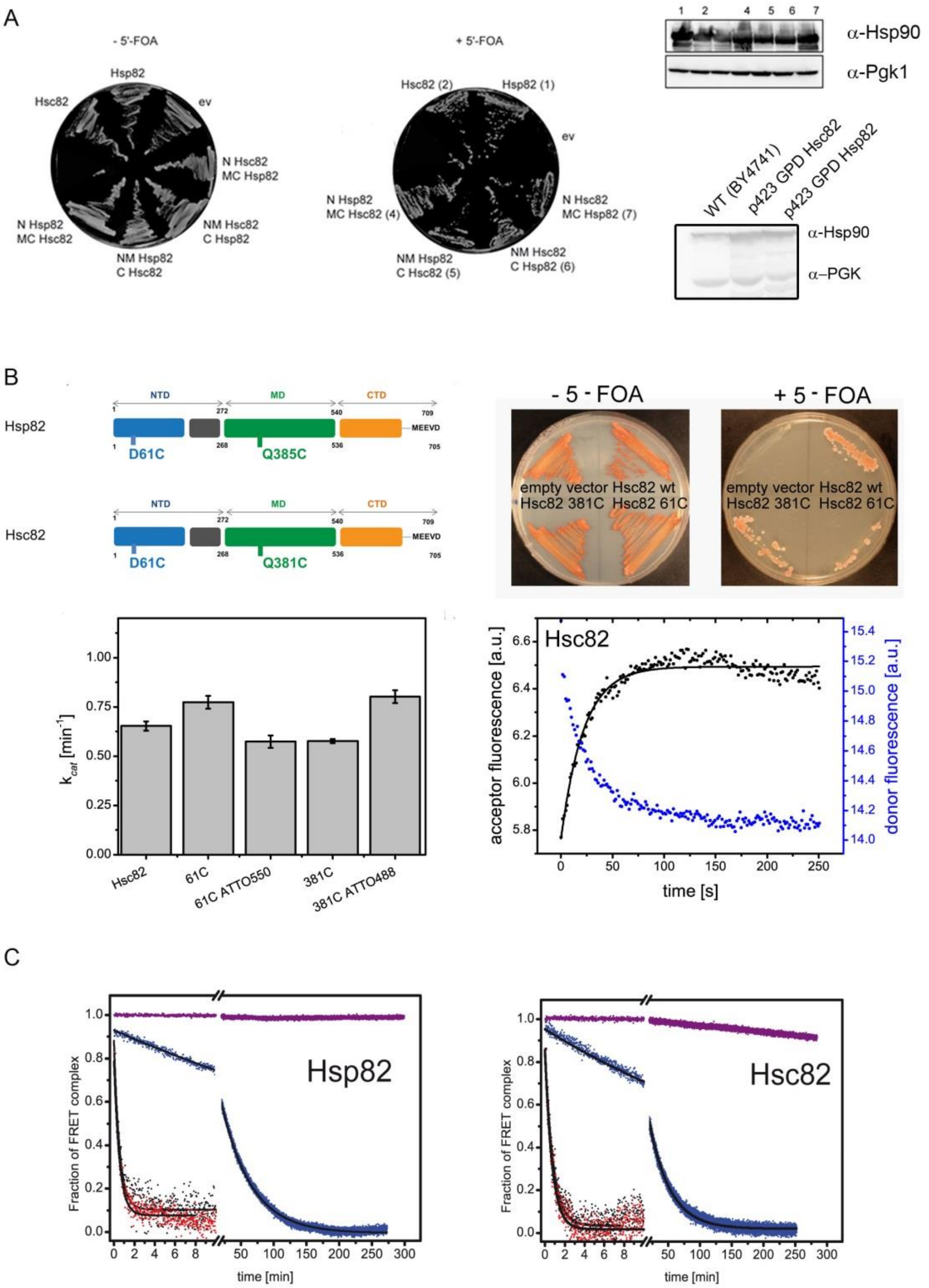
A
- 5'-FOA
Hsp82
Hsc82
ev
N Hsc82
MC Hsp82
N Hsp82
MC Hsc82
NM Hsc82
C Hsp82
NM Hsp82
C Hsc82
+ 5'-FOA
Hsc82 (2)
Hsp82 (1)
ev
N Hsp82
MC Hsc82 (4)
N Hsc82
MC Hsp82 (7)
NM Hsp82
C Hsc82 (5)
NM Hsc82
C Hsp82 (6)
1 2 4 5 6 7
α-Hsp90
α-Pgk1
WT (BY4741)
p423 GPD Hsc82
p423 GPD Hsp82
α-Hsp90
α-PGK
B
Hsp82
Hsc82
NTD
MD
CTD
MEEVD
D61C
Q385C
Q381C
- 5 - FOA
+ 5 - FOA
empty vector Hsc82 wt
Hsc82 381C Hsc82 61C
k_cat [min^-1]
Hsc82
61C
61C ATTO550
381C
381C ATTO488
acceptor fluorescence [a.u.]
donor fluorescence [a.u.]
time [s]
C
Fraction of FRET complex
time [min]
Hsp82
Hsc82

Supplementary Fig. 3: Growth and FRET control experiments. **A**: **Left panel**: Viability analysis and expression level of the different Hsp90 isoform constructs. Viability was tested by a 5-FOA plasmid shuffling approach. ev: control experiment with an empty vector. **Upper right panel:** Expression level of the respective constructs was monitored by a Western Blot. The enumeration of the constructs in the viability assay (left side) and the Western blot is identical. α-Pgk1 was used for the loading control**. Lower right panel:** Comparison of the expression levels of the overexpressed isoforms at 30 °C (Hsp82 and Hs82 expressed from a pGPD 423 vector) with the WT (total Hsp90 level in a BY 4741 strain, which was used for all *in vivo* experiments) **B:** FRET control experiments**. Upper left panel**: Scheme depicting the cysteine mutants used to perform FRET experiments with Hsp82 and Hsc82. Residues that are changed to cysteines (used for labelling the proteins with ATTO 488) are indicated. **Upper right panel:** Viability of Hsc82 C61 and Hsc82 C381 was tested by a 5-FOA plasmid shuffling approach. **Lower left panel:** ATPase activities of Hsc82 cysteine mutants with and without ATTO dyes compared to wild-type Hsc82. Means and standard deviations were calculated from at least three technical replicates. All values were corrected for background activity by inhibiting the ATPase with RD**. Lower right panel:** Dimer-formation of donor- and acceptor-labeled Hsc82 followed by FRET. **C:** The stability of the closed states of Hsp82 and Hsc82 was investigated by a FRET chase experiment. The chase was induced by adding a 10-fold excess of unlabeled protein (Hsp82 or Hsc82) to a preformed FRET-complex (of Hsp82 or Hsc82) in the absence of nucleotide (black), in the presence of 2 mM ATP (red), 2 mM ATPγS (blue) or 2 mM AMP-PNP (purple).

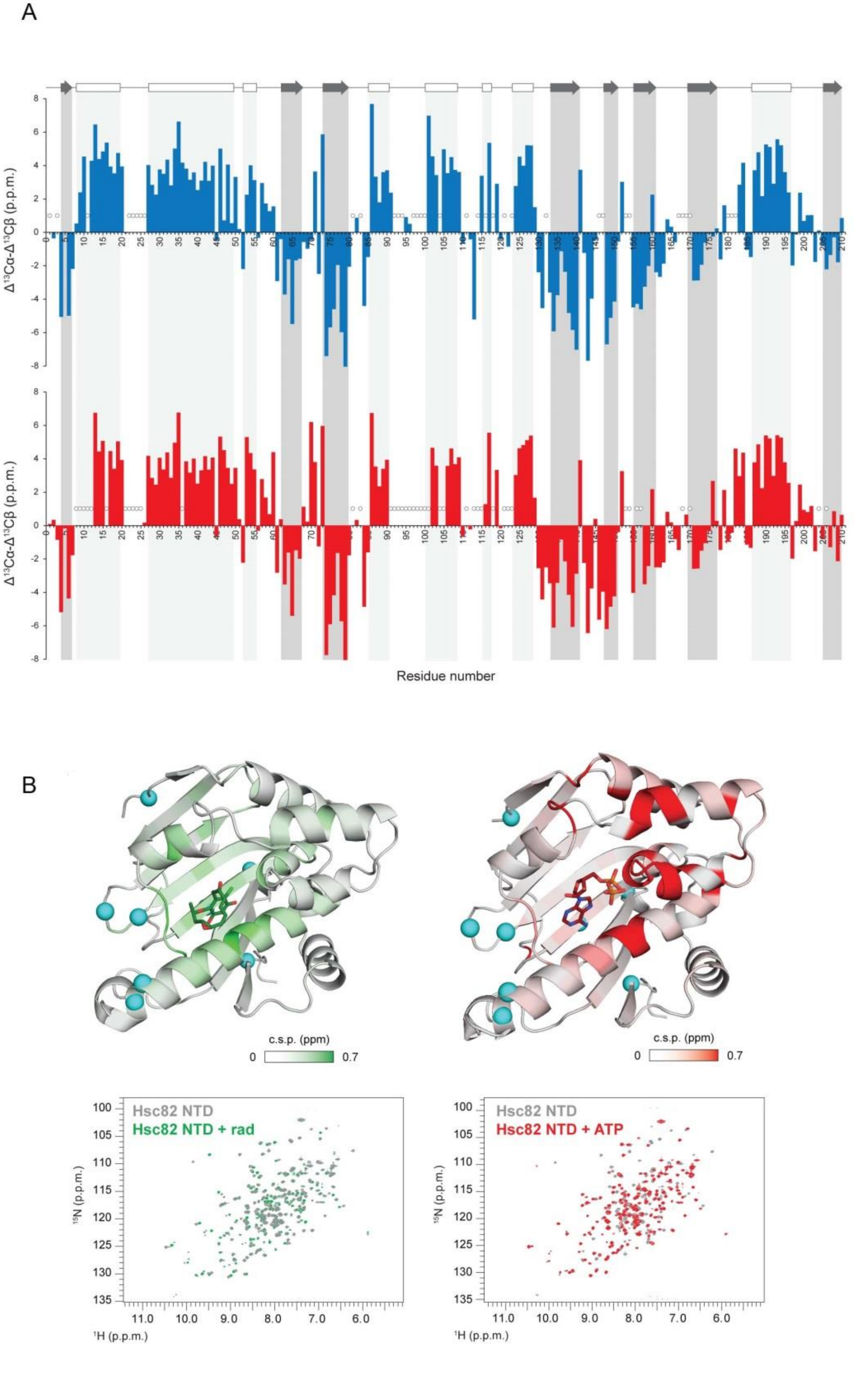

A
Δ13Cα-Δ13Cβ (p.p.m.)
Residue number
B
c.s.p. (ppm)
0
0.7
Hsc82 NTD
Hsc82 NTD + rad
Hsc82 NTD + ATP
15N (p.p.m.)
1H (p.p.m.)

Supplementary Fig. 4: NMR analysis of Hsp82 and Hsc82. A: ^{13}C secondary chemical shifts of Hsc82 NTD (blue) and Hsp82 (red) as a function of the residue number. No significant differences between the secondary structures of the two isoforms are seen. Secondary chemical shifts were calculated for each residue as the difference between the experimental ^{13}C shift and its random coil shift using CcpNmr Analysis[2]: positive values indicate α-helical conformation, negative β-strand and zero values unstructured regions. Structural elements extracted from TALOS+ software[3] are shown on top of each plot: arrows represent β-strands, rectangles α-helices. Empty dots indicate residues without data. **B:** Effects of RD and ATP binding to the NTDs of Hsp82/Hsc82 analyzed by NMR spectroscopy**.** Left panel: mapping of NMR chemical shift perturbation (CSP) of Hsc82 NTD upon RD binding onto the crystal structure of Hsp82 NTD bound to RD (PDB id 1BGQ[4]). Right panel: mapping of CSPs of Hsc82 NTD upon ATP binding onto the crystal structure of Hsp82 NTD bound to ATP (PDB id 1AM1[5]). In both cases, ligands are shown by sticks colored in dark green or red (for RD or ATP respectively) and changed residues are represented by cyan spheres. The overlay of the ^{1}H-^{15}N HSQC spectra corresponding to the apo- and bound-forms are shown at the bottom.

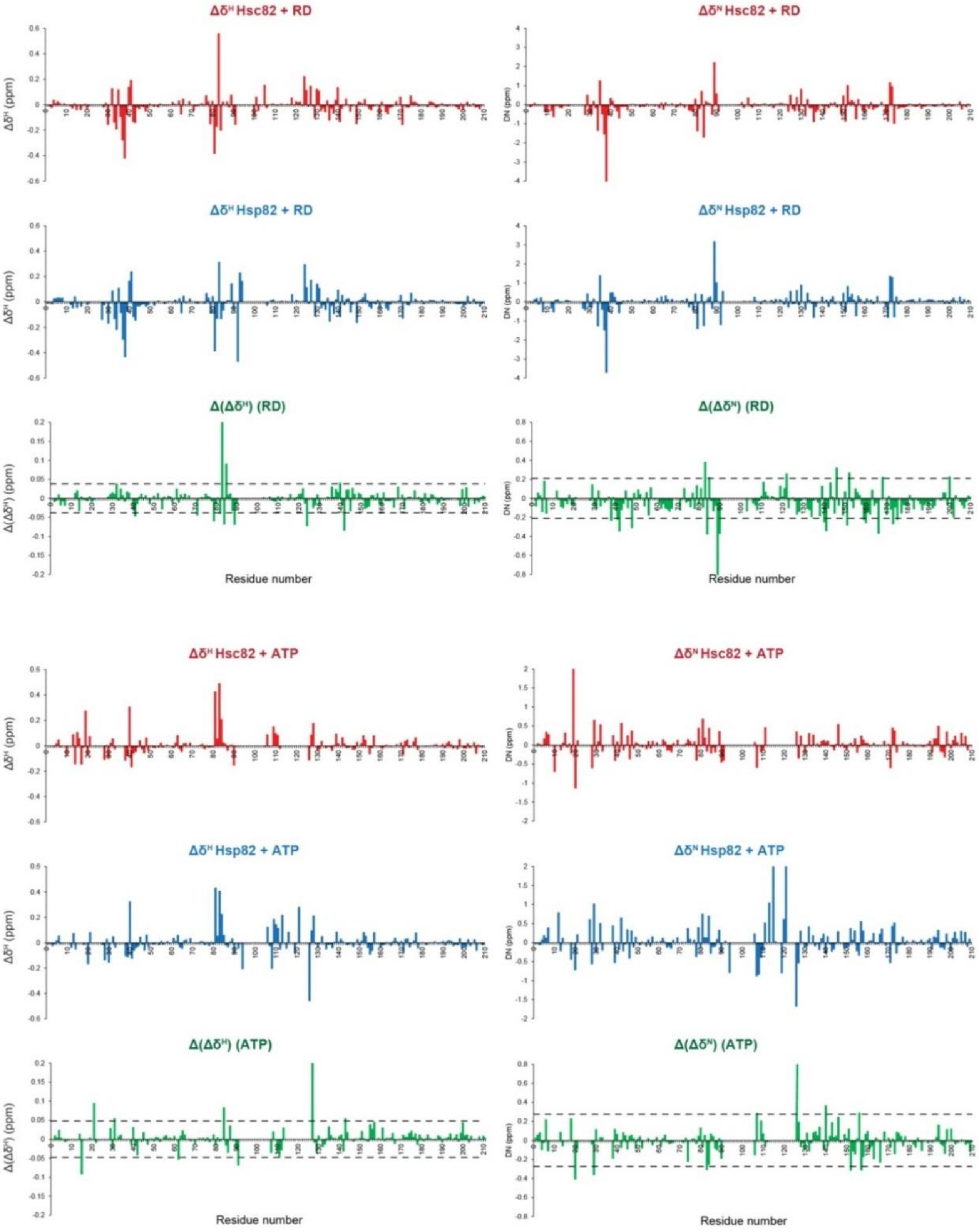
Δδ^H Hsc82 + RD
Δδ^N Hsc82 + RD
Δδ^H Hsp82 + RD
Δδ^N Hsp82 + RD
Δ(Δδ^H) (RD)
Δ(Δδ^N) (RD)
Δδ^H Hsc82 + ATP
Δδ^N Hsc82 + ATP
Δδ^H Hsp82 + ATP
Δδ^N Hsp82 + ATP
Δ(Δδ^H) (ATP)
Δ(Δδ^N) (ATP)
Δδ^H (ppm)
DN (ppm)
Δ(Δδ^H) (ppm)
Residue number

Supplementary Fig. 5: NMR data in presence RD and ATP. Differences in the ^{1}H and ^{15}N chemical shifts ($\Delta\delta^{X} = \delta^{X, BOUND} - \delta^{X, APO}$) upon binding of RD (upper panel) or ATP (lower panel), for Hsc82 (red) and Hsp82 (blue). The differential values, $\Delta(\Delta\delta^{X}) = \Delta\delta^{X, HSC82} - \Delta\delta^{X, HSP82}$, are plotted below as a function of the residue number (green). The residues for which $\Delta(\Delta\delta^{X})$ is above the mean + standard deviation (dotted line) are assumed to differ significantly between isoforms upon RD/ATP binding.

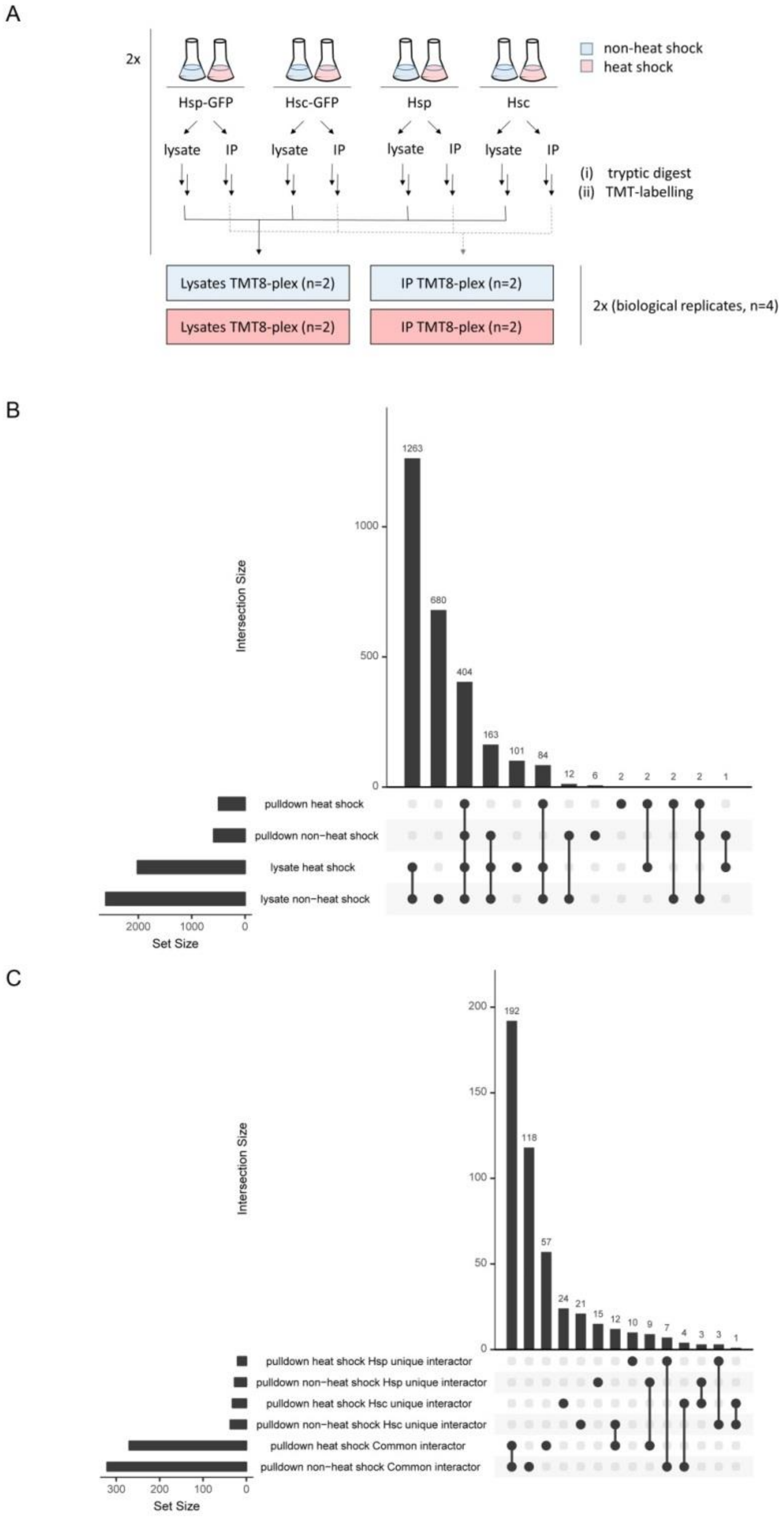
A
2x
non-heat shock
heat shock
Hsp-GFP
Hsc-GFP
Hsp
Hsc
lysate
IP
(i) tryptic digest
(ii) TMT-labelling
Lysates TMT8-plex (n=2)
IP TMT8-plex (n=2)
Lysates TMT8-plex (n=2)
IP TMT8-plex (n=2)
2x (biological replicates, n=4)
B
Intersection Size
1263
680
404
163
101
84
12
6
2
2
2
2
1
pulldown heat shock
pulldown non−heat shock
lysate heat shock
lysate non−heat shock
Set Size
C
Intersection Size
192
118
57
24
21
15
12
10
9
7
4
3
3
1
pulldown heat shock Hsp unique interactor
pulldown non−heat shock Hsp unique interactor
pulldown heat shock Hsc unique interactor
pulldown non−heat shock Hsc unique interactor
pulldown heat shock Common interactor
pulldown non−heat shock Common interactor
Set Size

Supplementary Fig. 6: Interactome analysis of the Hsp90 isoforms. A: Cartoon depicting the experimental flow of the experiment. Duplicates of the pulldowns with the GFP-tagged isoforms and their matched controls (samples with untagged isoforms) were simultaneously analyzed in one MS run. Two runs of each experiment were performed, such that in total quadruplicates of each sample were analyzed. Lysates were analyzed in an identical manner to the pulldown experiments. The total experiment was performed twice (under non-heat shock and heat-shock conditions). **B:** UpSetR plot showing the distribution of identified proteins. **C:** The different interactor categories (common, Hsp unique and Hsc unique), their sizes and their overlaps under non-heat shock conditions are shown with an UpSetR plot.

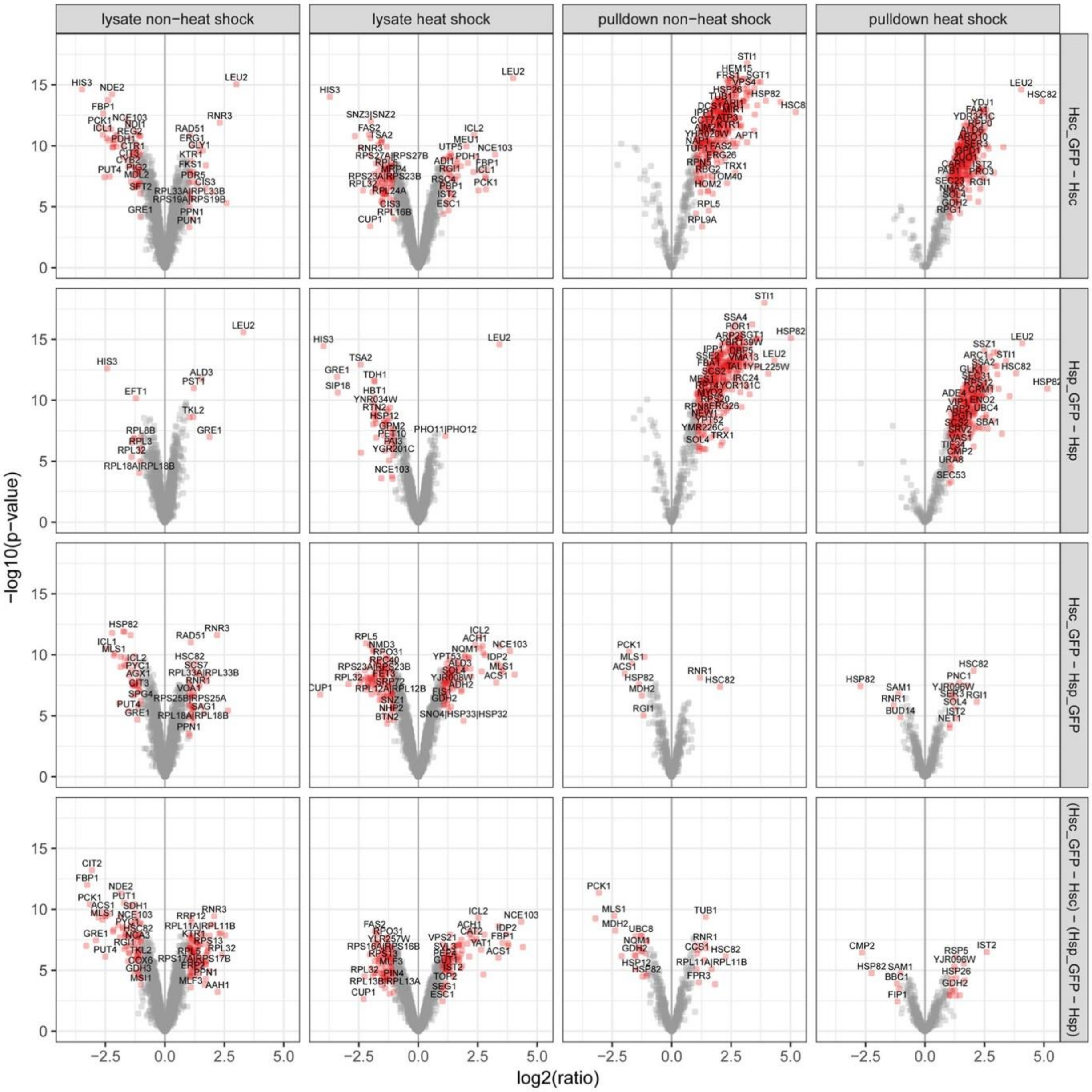

Supplementary Fig. 7: Volcano plots. Data for lysates and pulldowns under non-heat shock and heat shock are shown. p-values and fold-changes were calculated with limma (limma uses a moderated t-test).

A

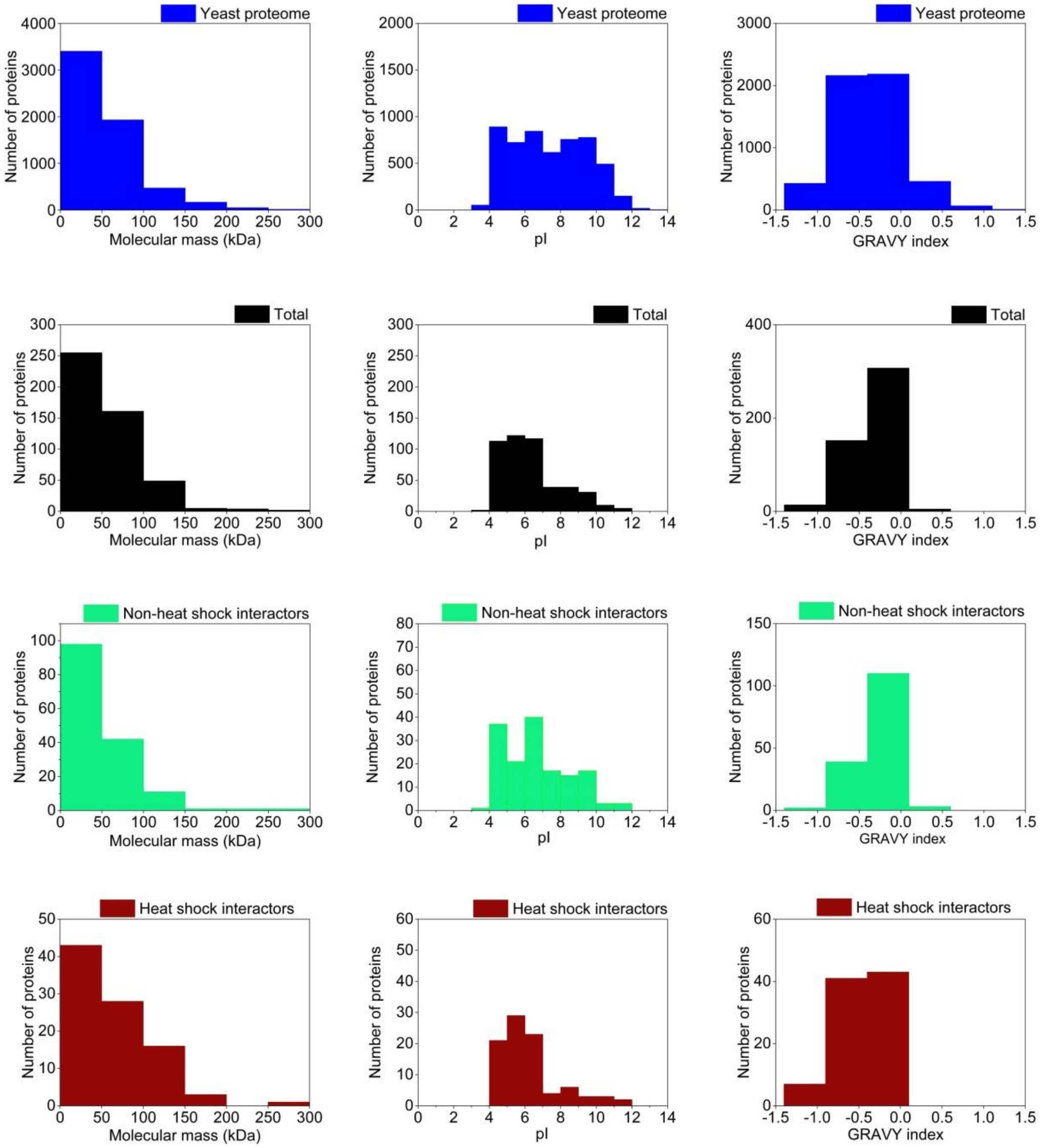

Yeast proteome
Total
Non-heat shock interactors
Heat shock interactors
Number of proteins
Molecular mass (kDa)
pI
GRAVY index

B

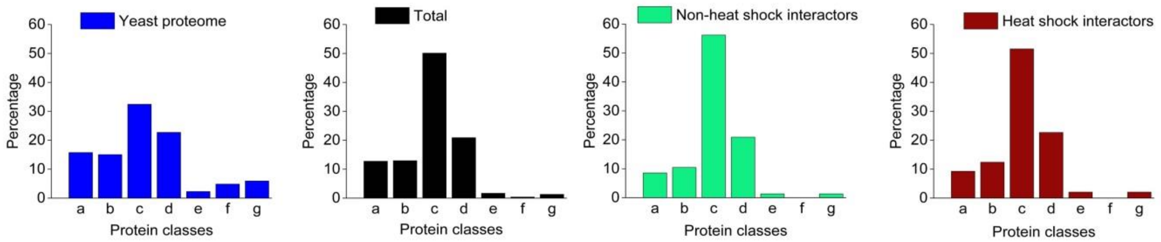
Yeast proteome
Total
Non-heat shock interactors
Heat shock interactors
Percentage
Protein classes
a b c d e f g
0 10 20 30 40 50 60

C

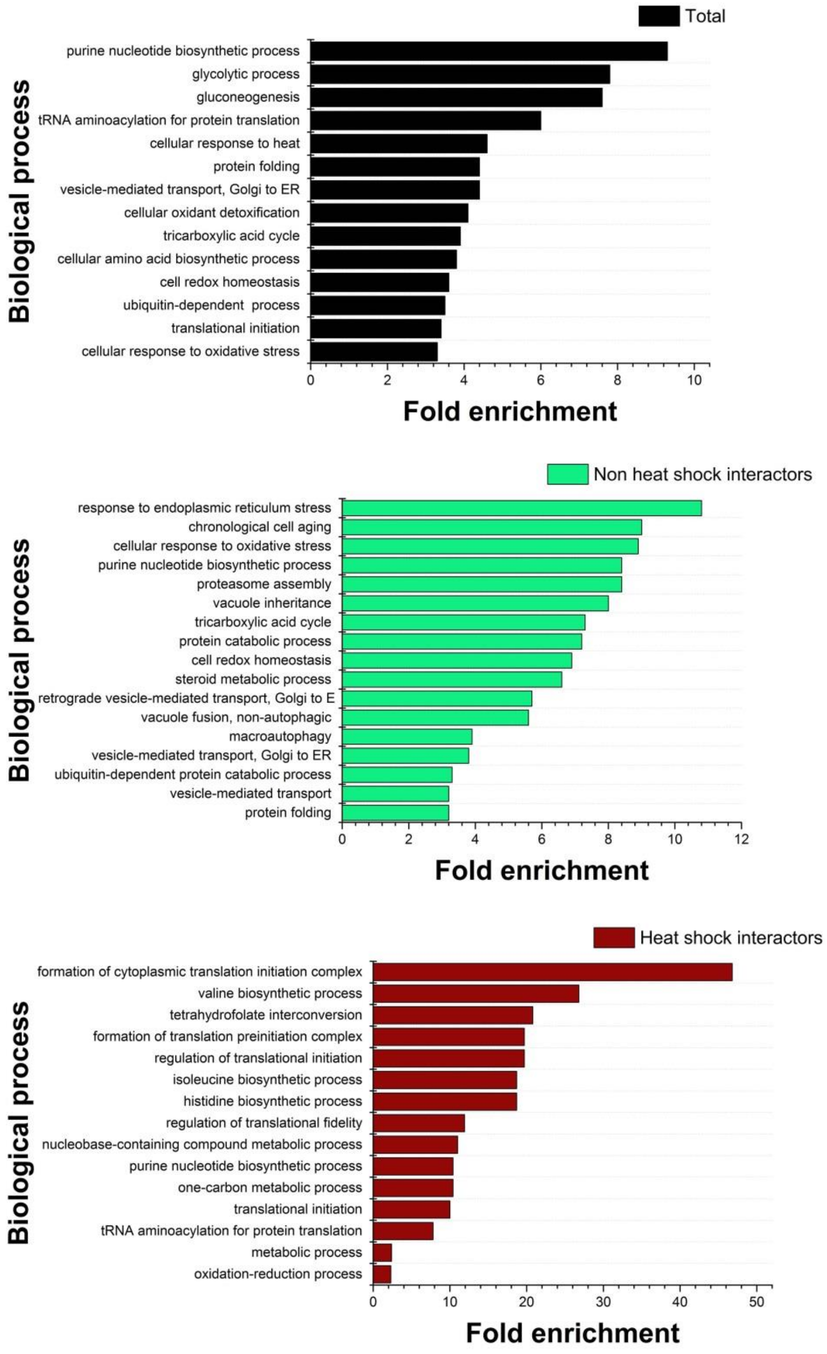
Total
Biological process
purine nucleotide biosynthetic process
glycolytic process
gluconeogenesis
tRNA aminoacylation for protein translation
cellular response to heat
protein folding
vesicle-mediated transport, Golgi to ER
cellular oxidant detoxification
tricarboxylic acid cycle
cellular amino acid biosynthetic process
cell redox homeostasis
ubiquitin-dependent process
translational initiation
cellular response to oxidative stress
Fold enrichment
0 2 4 6 8 10
Non heat shock interactors
Biological process
response to endoplasmic reticulum stress
chronological cell aging
cellular response to oxidative stress
purine nucleotide biosynthetic process
proteasome assembly
vacuole inheritance
tricarboxylic acid cycle
protein catabolic process
cell redox homeostasis
steroid metabolic process
retrograde vesicle-mediated transport, Golgi to E
vacuole fusion, non-autophagic
macroautophagy
vesicle-mediated transport, Golgi to ER
ubiquitin-dependent protein catabolic process
vesicle-mediated transport
protein folding
Fold enrichment
0 2 4 6 8 10 12
Heat shock interactors
Biological process
formation of cytoplasmic translation initiation complex
valine biosynthetic process
tetrahydrofolate interconversion
formation of translation preinitiation complex
regulation of translational initiation
isoleucine biosynthetic process
histidine biosynthetic process
regulation of translational fidelity
nucleobase-containing compound metabolic process
purine nucleotide biosynthetic process
one-carbon metabolic process
translational initiation
tRNA aminoacylation for protein translation
metabolic process
oxidation-reduction process
Fold enrichment
0 10 20 30 40 50

Supplementary Fig. 8: Biophysical characteristics, protein classes and enriched GO terms. A: Comparison of the molecular mass, the isoelectric point (pI) and the overall hydrophobicity (GRAVY score). The total interactors (black), the non-heat shock interactors (green) and heat shock interactors (red) were compared to the yeast proteome (blue). **B:** Comparison of the SCOPe protein classes. Class distributions were determined using the information from the SCOPe 2.07 database. Colors are the same as in Supplementary Fig. 8A. **C:** Enriched biological processes. GO term analysis was performed using the information from the DAVID bioinformatics resource. Biological processes that are enriched more than 3-fold over the yeast proteome and comprise at least 2 % of all input proteins are shown. An EASE score of 0.1 was used as a threshold. Colors are the same as used in Supplementary Fig. 8A.

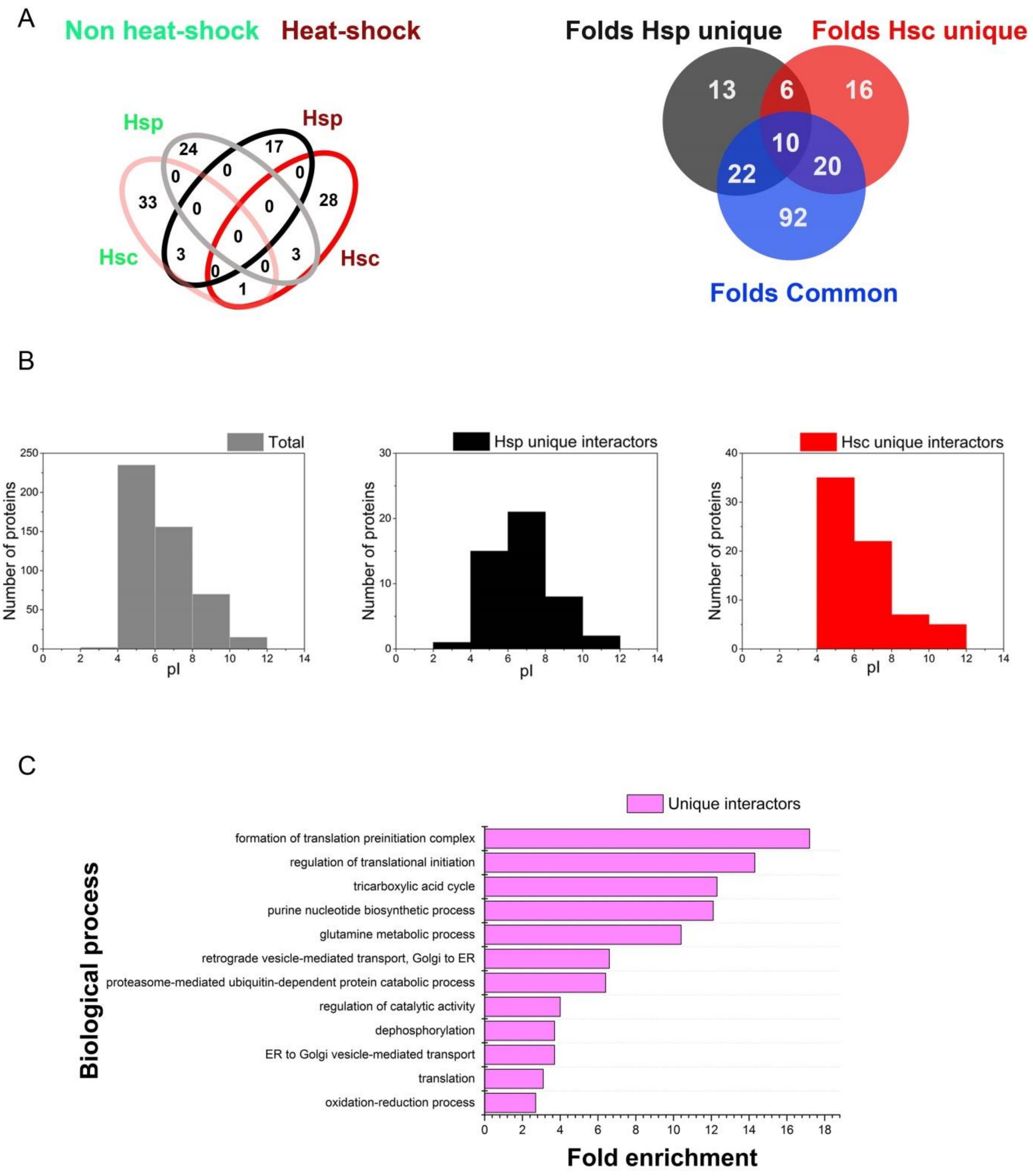
A
Non heat-shock
Heat-shock
Hsp
Hsc
Hsp
Hsc
24
17
33
28
Folds Hsp unique
Folds Hsc unique
Folds Common
13
6
16
10
22
20
92
B
Total
Hsp unique interactors
Hsc unique interactors
Number of proteins
pI
C
Unique interactors
Biological process
formation of translation preinitiation complex
regulation of translational initiation
tricarboxylic acid cycle
purine nucleotide biosynthetic process
glutamine metabolic process
retrograde vesicle-mediated transport, Golgi to ER
proteasome-mediated ubiquitin-dependent protein catabolic process
regulation of catalytic activity
dephosphorylation
ER to Golgi vesicle-mediated transport
translation
oxidation-reduction process
Fold enrichment

Supplementary Fig. 9: Unique interactors. A: Left panel: Overlap of the unique interactors under non-heat shock and heat shock conditions (also shown in Fig. 6C) in more detail. Three proteins that were classified as Hsp unique interactors under non-heat shock switch and become Hsc unique interactors under heat shock and vice versa. Only one protein is classified as Hsc unique interactor under non-heat shock and heat shock conditions (Ssp120, Supplementary Data 2). **Right panel:** Overlap of SCOPe folds of the unique interactors and the common interactors. SCOPe folds were assigned using the information from the SCOPe 2.07 database. **B:** Biophysical characteristics of the Hsp unique interactors and Hsc unique interactors compared to the total interactors. **C:** GO direct term analysis (of the biological processes) of all unique interactors. The GO term enrichment analysis was performed with the thresholds described in Supplementary Fig. 8C.

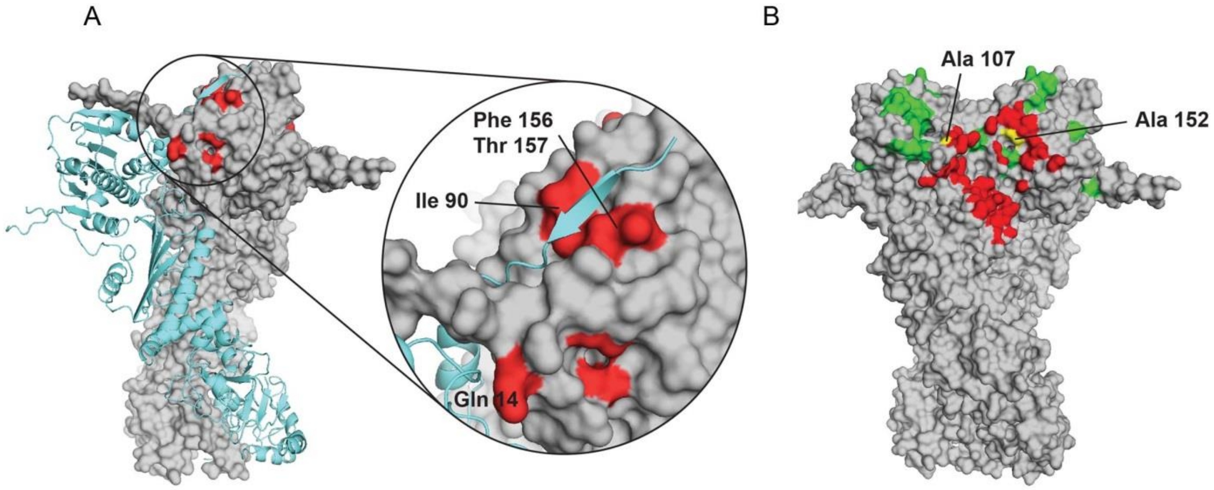

Supplementary Fig. 10: Cartoons depicting findings from the NMR analysis. A: Surface representation of the X-ray structure of Hsp82 in the closed conformation (PDB id 2CG9[6]) showing residues of the NTD that have a different response upon ATP binding between isoforms (red). One protomer is represented as cartoon for clarity. The inset shows a zoomed view of the interprotomer contacts in NTD**. B:** Surface representation of the same structure showing residues of the NTD that experience high CSP between isoforms or are differently affected upon ATP binding (green) together with the binding surface of the co-chaperone p23/Sba1 (red[6]). As seen, two residues that show high CSPs between isoforms or perturb differently upon ATP binding are located within the p23/Sba1 binding site (overlapping residues are shown in yellow).

Nr.	Vector	Insert	Construct obtained
1	pET-28 a (+)	6His_Sumo	pET28_6His_Sumo
2	pET28_6His_Sumo	Hsp82	pET28_6His_Sumo_Hsp82
3	pET28_6His_Sumo	Hsc82	pET28_6His_Sumo_Hsc82
4	p423 GPD	Hsp82	p423 GPD_Hsp82
5	p423 GPD	Hsc82	p423 GPD_Hsc82
6	p423 GPD	N_Hsp82_MC_Hsc82	p423 GPD_N_Hsp82_NM_Hsc82
7	p423 GPD	NM_Hsp82_C_Hsc82	p423 GPD_NM_Hsp82_C_Hsc82
8	p423 GPD	N_Hsc82_MC_Hsp82	p423 GPD_N_Hsc82_MC_Hsp82
9	p423 GPD	NM_Hsc82_C_Hsp82	p423 GPD_NM_Hsc82_C_Hsp82
10	p425 GPD	Hsp82_GFP	P425 GPD_Hsp82_GFP
11	P425 GPD	Hsc82_GFP	P425 GPD_Hsc82_GFP

Nr.	Fwd primer(s)	Reverse primer(s)
1	ctggtgccgcgcggcagccatatgatgggcagccatcaccatcac	gtcgacggagctcgaattcggatcctgcggccgccagtgtgat
2	tcacagagaacagattggtggatccatggctagtgaaacttttg	agtggtggtggtggtggtgctcgagctaatctacctcttccatttc
3	tcacagagaacagattggtggatccatggctggtgaaacttttg	agtggtggtggtggtggtgctcgagttaatcaacttcttccatctc
4	cgacggatctagaactagtggatccatggctagtgaaacttttg	actaattacatgactcgaggtcgacctaatctacctcttccatttc
5	cgacggatctagaactagtggatccatggctggtgaaacttttg	actaattacatgactcgaggtcgacttaatcaacttcttccatctc
6	cgacggatctagaactagtgatggctagtgaaacttttg	atggcttagtttgtttagttcttctatctcttg
	aactaaacaaactaagccattatggactag	actaattacatgactcgaggttaatcaacttcttccatctc
7	cgacggatctagaactagtgatggctagtgaaacttttg	tccttctctctctttcagctttttcttcg
	agctgaaagagagaaggagatcaaag	actaattacatgactcgaggttaatcaacttcttccatctc
8	cgacggatctagaactagtggatccatggctggtgaaacttttg	aaggcttagtcttgttcaactcttctaattcttg
	gttgaacaagactaagcctttgtggactag	actaattacatgactcgaggtcgacctaatctacctcttccatttc
9	cgacggatctagaactagtggatccatggctggtgaaacttttg	tctccttctctctttcagctttttcttcg
	agctgaaagagagaaggagatcaaagaatatg	actaattacatgactcgaggtcgacctaatctacctcttccatttc
10	cgacggatctagaactagtggatcccgtacgctgcaggtcgac	cactagccatttatttgtacaattcatccataccatggg
	gtacaaataaatggctagtgaaacttttg	actaattacatgactcgaggtcgacctaatctacctcttccatttc
11	cgacggatctagaactagtggatccatggctggtgaaacttttg	gcagcgtacgttaatcaacttcttccatctc
	agttgattaacgtacgctgcaggtcgac	actaattacatgactcgaggtcgacttatttgtacaattcatccataccatggg

Supplementary Table 1: Table of constructs cloned for this work (upper table) and PCR primers used to obtain these constructs (lower table). For the constructs used for protein expression a 6His_Sumo tag (referred to as 6His_Sumo) was cloned into a pET28 a (+) plasmid first, which was then used for cloning of the pET28_6His_Sumo_Hsp90 constructs. Primers were designed using the NEBuilder Assembly Tool.

SUPPLEMENTARY REFERENCES:

1 Prodromou, C. *et al.* Identification and structural characterization of the ATP/ADP-binding site in the Hsp90 molecular chaperone. *Cell* **90**, 65-75 (1997).

2 Vranken, W. F. *et al.* The CCPN data model for NMR spectroscopy: Development of a software pipeline. *Proteins: Structure, Function, and Bioinformatics* **59**, 687-696, doi:10.1002/prot.20449 (2005).

3 Shen, Y., Delaglio, F., Cornilescu, G. & Bax, A. TALOS+: a hybrid method for predicting protein backbone torsion angles from NMR chemical shifts. *Journal of Biomolecular NMR* **44**, 213-223, doi:10.1007/s10858-009-9333-z (2009).

4 Roe, S. M. *et al.* Structural Basis for Inhibition of the Hsp90 Molecular Chaperone by the Antitumor Antibiotics Radicicol and Geldanamycin. *Journal of Medicinal Chemistry* **42**, 260-266, doi:10.1021/jm980403y (1999).

5 Prodromou, C. *et al.* Identification and Structural Characterization of the ATP/ADP-Binding Site in the Hsp90 Molecular Chaperone. *Cell* **90**, 65-75, doi:10.1016/S0092-8674(00)80314-1.

6 Ali, M. M. *et al.* Crystal structure of an Hsp90-nucleotide-p23/Sba1 closed chaperone complex. *Nature* **440**, 1013-1017, doi:10.1038/nature04716 (2006).
