# Supplementary material for: The Hsp90 isoforms from S. cerevisiae differ in structure, function and client range
Source: Nat Commun. 2019 Aug 9;10:3626. doi: 10.1038/s41467-019-11518-w (PMC6689086; doi:10.1038/s41467-019-11518-w)
Supplement: Supplementary file 2 — Description of Additional Supplementary Files [file 41467_2019_11518_MOESM2_ESM.pdf]

Description of Additional Supplementary Files

File name: Supplementary Data 1

Description: MS analysis of lysates and pulldowns: Supplementary Data 1 shows a summary of the mass spectrometry results as well as the statistical output of the differential abundance test using limma.

File name: Supplementary Data 2

Description: Interactors of the Hsp90 isoforms identified in the pulldown experiments. Interactors are listed and the respective category (common, Hsp unique, Hsc unique) is indicated. The table also contains the biophysical parameters (molecular mass, pI, GRAVY score) and SCOPe folds of the interactors.

File name: Supplementary Data 3

Description: SCOPe folds of the interactors. Enriched SCOPe folds were assigned using the information from the SCOPe 2.07 database. Folds that were found at least twice and are enriched >1 compared to the yeast proteome are listed for the total interactors, non-heat shock interactors and heat-shock interactors. The significance of the enrichment was determined using Fisher's exact test. For the unique interactors folds that were only found in the categories "Hsp unique interactors" and Hsc unique interactors" are listed.
