# Supplementary material for: The Hsp90 isoforms from S. cerevisiae differ in structure, function and client range
Source: Nat Commun. 2019 Aug 9;10:3626. doi: 10.1038/s41467-019-11518-w (PMC6689086; doi:10.1038/s41467-019-11518-w)
Supplement: Supplementary file 6 — Reporting Summary [file 41467_2019_11518_MOESM6_ESM.pdf]

nature research

Corresponding author(s): Johannes Buchner

Last updated by author(s): YYYY-MM-DD

Reporting Summary

Nature Research wishes to improve the reproducibility of the work that we publish. This form provides structure for consistency and transparency in reporting. For further information on Nature Research policies, see Authors & Referees and the Editorial Policy Checklist.

Statistics

For all statistical analyses, confirm that the following items are present in the figure legend, table legend, main text, or Methods section.

n/a	Confirmed	
☐	☒	The exact sample size (*n*) for each experimental group/condition, given as a discrete number and unit of measurement
☐	☒	A statement on whether measurements were taken from distinct samples or whether the same sample was measured repeatedly
☐	☒	The statistical test(s) used AND whether they are one- or two-sided<br>*Only common tests should be described solely by name; describe more complex techniques in the Methods section.*
☐	☒	A description of all covariates tested
☐	☒	A description of any assumptions or corrections, such as tests of normality and adjustment for multiple comparisons
☐	☒	A full description of the statistical parameters including central tendency (e.g. means) or other basic estimates (e.g. regression coefficient) AND variation (e.g. standard deviation) or associated estimates of uncertainty (e.g. confidence intervals)
☐	☒	For null hypothesis testing, the test statistic (e.g. *F*, *t*, *r*) with confidence intervals, effect sizes, degrees of freedom and *P* value noted<br>*Give P values as exact values whenever suitable.*
☒	☐	For Bayesian analysis, information on the choice of priors and Markov chain Monte Carlo settings
☒	☐	For hierarchical and complex designs, identification of the appropriate level for tests and full reporting of outcomes
☒	☐	Estimates of effect sizes (e.g. Cohen's *d*, Pearson's *r*), indicating how they were calculated

Our web collection on statistics for biologists contains articles on many of the points above.

Software and code

Policy information about availability of computer code

Data collection	IsobarQuant and Mascot (v2.2.07) were used to process the acquired raw mass spec data.
Data analysis	The R programming language was used to analyze the raw output data of IsobarQuant as described in the Method section of the manuscript in the section "Data analysis".<br>GO direct terms were assigned using the DAVID bioinformatics resource. Venn diagrams were created with the help of http://bioinfogp.cnb.csic.es/tools/venny/. SCOPe folds were assigned with http://phosphatome.net/2.0/blast-scop.

For manuscripts utilizing custom algorithms or software that are central to the research but not yet described in published literature, software must be made available to editors/reviewers. We strongly encourage code deposition in a community repository (e.g. GitHub). See the Nature Research guidelines for submitting code & software for further information.

Data

Policy information about availability of data

All manuscripts must include a data availability statement. This statement should provide the following information, where applicable:

- Accession codes, unique identifiers, or web links for publicly available datasets
- A list of figures that have associated raw data
- A description of any restrictions on data availability

All data generated or analyzed during this study are included in this published article (and its supplementary information files).
The raw mass spec data will be uploaded to PRIDE.

Field-specific reporting

Please select the one below that is the best fit for your research. If you are not sure, read the appropriate sections before making your selection.

☒ Life sciences ☐ Behavioural & social sciences ☐ Ecological, evolutionary & environmental sciences

For a reference copy of the document with all sections, see nature.com/documents/nr-reporting-summary-flat.pdf

Life sciences study design

All studies must disclose on these points even when the disclosure is negative.

Sample size	We used the same yeast strain background for the experiment (because strains were created via a plasmid shuffling approach) and therefore, expected low variability between replicates. Furthermore, due to financial and workforce constraints, we performed four replicates.
Data exclusions	we did not exclude any data
Replication	We only kept data that was reproducible in all the four yeast strain replicates used.
Randomization	n/a
Blinding	n/a

Reporting for specific materials, systems and methods

We require information from authors about some types of materials, experimental systems and methods used in many studies. Here, indicate whether each material, system or method listed is relevant to your study. If you are not sure if a list item applies to your research, read the appropriate section before selecting a response.

Materials & experimental systems

n/a	Involved in the study	
☐	☒	Antibodies
☒	☐	Eukaryotic cell lines
☒	☐	Palaeontology
☒	☐	Animals and other organisms
☒	☐	Human research participants
☒	☐	Clinical data

Methods

n/a	Involved in the study	
☒	☐	ChIP-seq
☒	☐	Flow cytometry
☒	☐	MRI-based neuroimaging

Antibodies

Antibodies used	anti-yeast Hsp90 polyclonal antibody (Pineda Antibody Service), anti-PGK1 monoclonal antibody (Novex, cat. no. A459250, clone 22C5D), anti-HA monoclonal antibody (Sigma, product number H9658, clone HA-7) and anti-GFP antibody (Roche, cat. no. 11814460001, from mouse IgG1κ (clones 7.1 and 13.1)), anti-rabbit IgG-peroxidase coupled antibodies (Sigma-Aldrich, cat. nos. A9044 and A0545)
Validation	The anti-yeast Hsp90 antibody was raised against His-Hsp82 from yeast that had been purified in our lab using recombinatn expression in E. coli. Antibody production was performed by Pineda Antibody Service (www. pineda-abservice.de) and the serum was tested in Western blot experiments using BY4741 yeast lysates. The pre-immune serum was used to control if there is any unspecific antibody binding.<br>The anti-PGK1 monoclonal antibody (Novex, cat. no. A459250) has been tested in Western blot experiment with yeast samples by Novex (Thermo Fisher) and has been used in 122 publications as stated on the manufacturer's website (https://www.thermofisher.com/antibody/product/PGK1-Antibody-clone-22C5D8-Monoclonal/459250).<br>The anti-HA monoclonal antibody (Sigma, product number H9658, clone HA-7) has been validated for use in immunoblotting: https://www.sigmaaldrich.com/content/dam/sigma-aldrich/docs/Sigma/Datasheet/7/h9658dat.pdf<br><br>Information about the anti-GFP antibody (Roche, cat. no. 11814460001, from mouse IgG1κ (clones 7.1 and 13.1) is given here: https://www.sigmaaldrich.com/catalog/product/roche/11814460001?lang=de&region=DE
